# Transport Personnel Health Cohort (TRAPHEAC): study protocol and methodological considerations

**DOI:** 10.1265/ehpm.25-00127

**Published:** 2025-07-19

**Authors:** Irina Guseva Canu, Viviane Fiona Mathilde Remy

**Affiliations:** Department of Occupational and Environmental Health, Unisanté, University of Lausanne, Switzerland

**Keywords:** Bus driver, Occupational exposure, Retrospective exposure assessment, Hospital discharge report, Patient-reported outcome measure, Bias

## Abstract

**Background:**

Only prospective cohort studies can capture changes in work conditions and their effects on health. Such studies are rare in bus drivers, despite their high rates of injuries and diseases. The three existing cohorts have limited exposure data, collected at baseline and thus uninformative on exposure and exposure-effect dynamics. Therefore, we aimed to develop the Swiss Transport Personnel Health Cohort (TRAPHEAC) and to anticipate and prevent potential bias.

**Methods:**

To set up the study protocol, we first organized the stakeholder consultation and available data inventory. Second, we mapped the exposure-outcomes pairs to list the most prevalent occupational hazards, and conducted exposure measurement campaigns. Third, we built the Swiss Bus-Exposure Matrix for physical-chemical hazards and Bus-Ergonomics Matrix for visual and biomechanical constrains. These matrices contain 705 bus models operated in Switzerland since 1980 and enable assessing current and past exposure when merged with bus drivers’ work histories.

**Results:**

We opted for an original study design combining prospective cohort part starting at 2024 and a retrospective part with nested case-control studies. Bus drivers will be invited through three complementary channels: unions, companies, and social media. The eligibility screening, information, and consent form signature and registration will be conducted using the study web-site modules. Registered bus drivers will first receive a comprehensive inclusion questionnaire, then a yearly follow-up questionnaire to assess and update the drivers’ work histories. Validated self-reported questionnaires will be used for assessing additional health outcomes (e.g., stress, sleep problems, musculoskeletal disorders, burnout) and individual, occupational and live-style related factors (e.g., personality, ICT use, physical activity). Hospital records (with diagnosed diseases, diagnosis dates and treatments) centralized since 2000 by the Swiss Federal Statics Office will be used for assessing disease prevalence, incidence and case-control status. Advanced statistical analysis will be conducted to address etiological and methodological questions (e.g., individual and joint causal effects of multiple exposures and exposure components; time-varying exposure and outcome variables and confounders mixtures).

**Conclusions:**

The yearly assessment of both exposure and health outcomes should enable capturing changes in work conditions and their effects on bus drivers’ health and well-being over time and facilitate the tailoring, implementation and evaluation of preventive interventions.

**Supplementary information:**

The online version contains supplementary material available at https://doi.org/10.1265/ehpm.25-00127.

## Background

Noxious nature of bus driver occupation was pointed out since 1953 [1]. Multiple hazards including poor cabin ergonomics, rotating shift patterns, inflexible running time, dense traffic, and violence from passengers coexist in bus drivers’ job and could lead to poor health outcomes. Bus drivers are at risk of musculoskeletal disorders, cardiovascular diseases including hypertension, metabolic and digestive diseases and stress-related disorders [2–9]. This situation, known for almost 70 years, does not seem to change. Researchers continue reporting worse health outcomes in bus drivers compared to general population and speculate on their causality, without having adequate data for investigation.

Interestingly, many changes have occurred in the public transport sector over last 10–20 years. This includes dedicated bus lanes, electrification and green technologies, introduction of high-capacity articulated and double-decker buses, driver assistance technology on vehicles, contactless and mobile ticketing, and pandemic-related enhanced hygiene measures. To tackle these changes and their health effects, prospective cohort studies are necessary. Besides an administrative Spanish cohort [10], three prospective cohorts of bus drivers have been created. The first is the Danish cohort (n = 2045), launched in 1978 [11] and discontinued since 1993. The second is the Taiwanese cohort (N = 916), launched in 2005 [8] and still active. The last is the Polish cohort (n = 292), launched in 2016 as part of a preventive program focused on cardiovascular risk factors [12]. Unfortunately, in all these cohorts, limited exposure data were collected at baseline and cannot inform on exposure and exposure-effect dynamics.

In Switzerland, bus drivers were identified at risk of mortality from lung cancer [13] and suicides [14] in the Swiss national cohort. No data on occupational exposures were available, but the role of occupational risk factors independently from socio-economic and environmental factors was confirmed in both outcomes [15–17]. Furthermore, a higher prevalence of mental and behavioral disorders, substance-related and addictive disorders and mood disorders was identified as additional suicide risk factor in Swiss bus drivers compared to other workers [18].

These findings motivated a collaboration with Swiss public transport unions and reuse of their data. In a 3-wave repeated cross-sectional study, we found that working days over 10 h represents the most tedious work condition [19]. We also found that neck and shoulder pain, sleep disorders, sick leaves, and accidents increased since 2010 and were associated with working conditions, and co-morbidity and that the SARS-CoV-2 pandemic had additional negative consequences [20]. Given the methodological limitations and subjective exposure assessment in this study, no causal interpretation of finding was possible. However, the need of a scientifically sound and methodologically strong cohort study appeared obvious.

The present article describes the protocol development, design and methodological features of the Swiss Transport Personnel Health Cohort study (TRAPHEAC) aimed at evaluating causal relationships between bus drivers’ exposure and health. Methodological features important for preventive purpose are also discussed.

## Methods

### Stakeholder consultation

As we aimed to capitalize on existing data and experience, stakeholder consultation was paramount. Contacts were first established by email or phone to arrange an online meeting. When necessary, a second meeting was organized in person. Most contacted stakeholders provided signed collaboration agreements, funding or in-kind contribution (Table 1).

**Table 1:** Description of contacted stakeholders

**Name**	**Reason for contact**	**Support received**
Federal Office of Transport	Supervision of public transport in Switzerland (safety, finance, infrastructure, and political frameworks); Registry of road accidents (since 2011); Funding and support for research	Accident statistics provision; Connection with other stakeholders; Funding; Signed partnership
Federal Roads Office	Responsible for road infrastructure; Registry of delivered and renewed driving licenses potentially useful for identifying and sampling professional bus drivers with valid license	Access to the registry but impossibility to distinguish professional bus drivers among people with valid bus driver license
Federal Social Insurance Office	Expertise on policies related to old-age, invalidity and the family; Centralization of all retirement contribution data potential helpful for reconstructing career paths of bus drivers	None, since occupation variable is unavailable in retirement contribution data
Federal Statistical Office	Registry of Swiss residents; Registry of Swiss companies; Registry of hospital discharge data (since 2000); Deterministic and probabilistic data linkage	Variable selection and extraction from different FSO registries, centralization of data from other offices and linkage with the cohort; Signed partnership
Federal Office for the Environment	Environmental exposure monitoring data and geodata; Method development for complex multi-exposure data modeling; Funding and support for research	Data provision on residential exposure to noise, outdoor air pollution, and electromagnetic fields; Funding; Signed partnership
Federal Office of Public Health	Monitoring of environmental radioactivity and radiation doses in Switzerland; Funding and support for research	Data provision on residential radon exposure; Signed partnership
Swiss National Accident Insurance Fund	Occupational exposure monitoring; Extensive records on work-related accidents, injuries, and illnesses; Policy implementation; Funding and support for research	Assistance for vibration monitoring in buses: device and manpower provision
National Institute for Cancer Epidemiology Research	Centralized registry of cancer (since 2018); cancer incidence rates per location and histological subtype; central contact point with cantonal cancer registries	Agreement in principle for case identification and data provision
Union des Transports Publics	Coordination with public transport companies; Access to industry data (bus fleet composition and technology)	Access to some data and technical expertise; Signed partnership
Unions: SEV, Sydicom and SSP	Bus driver representation; Occupational health protection and promotion; Work condition improvement; Communication	Contact with affiliated bus drivers; Co-construction of study protocol and instruments; Signed partnerships
Certified medical centers and physicians	Conduct of standardized medical exam for driving fitness validation (compulsory every 5 years for driving license renewal); Potentially structured medica data and exam conclusion	None, due to lack of medical record harmonization and centralization

### Hazard identification and exposure assessment

As point of departure, we used the mind map of the bus drivers’ exposure and potentially related health outcomes, based on the literature review [19]. Both traditional and emergent (i.e., recently appeared and/or insufficiently known) hazards were considered. The latter included ultrafine particles (UFP), light-emitting diodes (LED) and electromagnetic fields (EMF). Regarding chemical, physical and biomechanical hazards as well as visual ergonomics (including LED on the bus dashboard) we assumed that exposure would be related to the bus model conception and technology. A three-step process was applied. First, we made an inventory of the bus models operated since 1980s and identified models representative of technological breakthroughs [21]. Second, standardized static and dynamic exposure measurement campaigns were conducted in each representative bus, in both urban and rural environments. This enabled assessing the Light and Visual contrast based on luminance (Mavo Spot 2, Gossen Germany), Air exchange rate (Testo 435, Testo AG, Germany), Equivalent noise and peak noise (B&K 4448, Bruel and Kjaer, Danemark), PM_10_ (pDR1500, Thermo Fisher Scientific, USA), UFP (Discmini, Testo AG, Germany), High and low frequency EMF (PMM 5083, PMM S.r.l, Italy), Vibrations (Nor136, Norsonic, Norway) and ergonomic parameters (measuring angle, dimensions and photo) as seven ergonomic scores: global, visual, biomechanic, shoulder girdle, upper body, lower body and back [22]. Finally, these data served to create a bus exposure matrix (BEM) for physical-chemical hazards [23] and a bus ergonomics matrix (BERM) for ergonomic contains [24]. Both matrices provide exposure levels to each hazard for 705 bus models. This enables the prospective and retrospective estimation of individual exposure by linking matrices with bus drivers’ history of driven bus models.

### Health outcome selection and measure

A cohort study enables investigating virtually all health outcomes provided the ability to measure them and sufficient statistical power. In TRAPHEAC, we decided to focus on 1-the most prevalent outcomes and 2-the less understood outcomes with respect to their causality and work-relatedness. Outcomes were classified either as diseases/disorders if medically established diagnosis is available (e.g., obstructive sleep apnea (ICD-11 code 7A41), intervertebral disc herniation of cervical region (ICD-11 code FA80.0), type 2 diabetes mellitus (ICD-11 code 5A12) or as subjectively measured outcome (e.g., burnout or perceived stress)). The latter will be measured at inclusion and annually, using the most validated instruments selected according to [25] and the Consensus-based Standards for the selection of health Measurement Instruments [26] (Table 2). With respect to perceived stress and its consequences, we distinguished between different sources of stress (occupational stress and work-family conflict *versus* general stress) and different outcomes (burnout *versus* depression). Indeed, the recognition of burnout as a stress-related health condition remains debated [41, 42] and there is evidence that burnout develops progressively, and in its severe, clinical form may evolve into depression [43, 44].

**Table 2:** Instruments for the assessment of study variable

**Instrument name and [Reference]**	**Description**	**Assessment period**
Bus-Exposure Matrix [23]	Database with quantitative exposure estimates (mean ± SD) for 10 physical and chemical hazards for 705 bus models used in Switzerland since 1980	Last 12 months and cumulated over 1985–2025 period
Bus-Ergonomics Matrix [24]	Database with ordinal scores (from 1 (worse) to 100 (best)) of ergonomics for five body parts: visual, shoulder girdle, upper body, back, and lower body, biomechanical, and global score for 705 bus models used in Switzerland since 1980	Last 12 months and cumulated over 1985–2025 period
Effort-Reward Imbalance Questionnaire (short version) [27]	16-items self-report measure assessing occupational stress by evaluating the imbalance between the effort invested at work and the rewards received in return.	Last 6 months
Perceived occupational stress scale [28]	4-items self-report measure rating a worker’s current perception of feeling stressed at work	No specified fixed timeframe
Job Stressfulness measure [29]	1-item self-report measure assessing perceived job stress	No specified fixed timeframe
Burnout Assessment Tool [30]	12-item self-report measure of current experience of burnout and core burnout symptoms (Exhaustion, Mental distance, Cognitive impairment, and Emotional Impairment)	No specified fixed timeframe
Beck Depression Inventory-II [31]	21-item self-report measure of depression aligned with DSM-IV diagnostic criteria divided in cognitive, affective somatic, and behavioral symptoms	Last two weeks
Borg Rating of Perceived Exertion [32]	1 item self-report measure of perceived level of physical effort during physical activity	Over a usual working day
Work-Family Conflict [33]	3-item self-report measure of the interference of work responsibilities with family life	No specified fixed timeframe
Perceived Stress Scale [34]	10-item self-report measure of general perception of stress and the feeling that life demands are overwhelming	Last month
Global Sleep Assessment Questionnaire [35]	10-item self-reported screening of 7 sleep disorders	No specified fixed timeframe
WHOQOL-BREF quality of life assessment [36]	26-item self-report measure of the quality of life, considering its physical, psychological, social, and environment-related domains	Last two weeks
International Physical Activity Questionnaire, Short version [37]	7-item self-report measure of the frequency and duration of physical activity at different intensities (vigorous, moderate, walking) as well as sedentary behavior	Last seven days
Big Five Inventory–2 extra-short form [38]	15-item self-report measure of general personality tendencies like extraversion, agreeableness, conscientiousness, negative emotionality, open-mindedness	No specified fixed timeframe
Medical history questionnaire [39]	Self-reported inventory of diseases diagnosed by a physician, diagnosis date, prescribe treatment and hospitalization organized by type of diseases (respiratory, digestive, cancer, etc.)	Lifespan
The Nordic Musculoskeletal Questionnaire [40]	Self-reported checklist of musculoskeletal symptoms in in the neck, shoulders, elbows, wrists/hands, upper back, low back, hips/thighs, knees, and ankles/feet	Last 12 months and Last seven days

### Other covariates

Inclusion and follow-up questionnaires will help collect data on additional work conditions (e.g., effort-reward imbalance, job stressfulness, perceived level of physical effort) and on individual data (e.g., personality, physical activity, quality of life) that may act as predictors, mediators, moderators or confounders in causal exposure-response analysis. These variables were selected based on the literature [19]. As for the outcome variables, the measurement instruments for these variables were selected according to [25, 26].

### Feasibility assessment and sample size estimation

Bus drivers’ potential willingness to participate in a future prospective cohort study and provide individual and medical data was assessed prior to the protocol development. For this we used a short survey nested in the previous cross-sectional study [20]. Out of 916 unionized bus drivers (response-rate 21%), 522 (57%) provided a complete work history and exploitable history of driven buses. Overall, 696 respondents (76%) reported wishing to participate; 568 (62%) would agree to provide access to their medical records; and (421) 46% would consent to provide the social insurance number for enabling further data linkage. Note that unionized drivers (targeted in this study) represent 40.3% of Swiss bus drivers, but unionization depends on the company, varying from 5% to 80%. This means that by opening the cohort to all (unionized and nonunionized) bus drivers and considering the same response-rate (as an optimistic hypothesis), 1740 participants would be expected at baseline. Under a pessimistic hypothesis of 5% response-rate in nonunionized bus drivers, the baseline sample should include 1023 participants. Such a cohort would thus have a comparable size as the Taiwanese cohort [3, 8].

## Results

### Study design

**Fig. 1:**
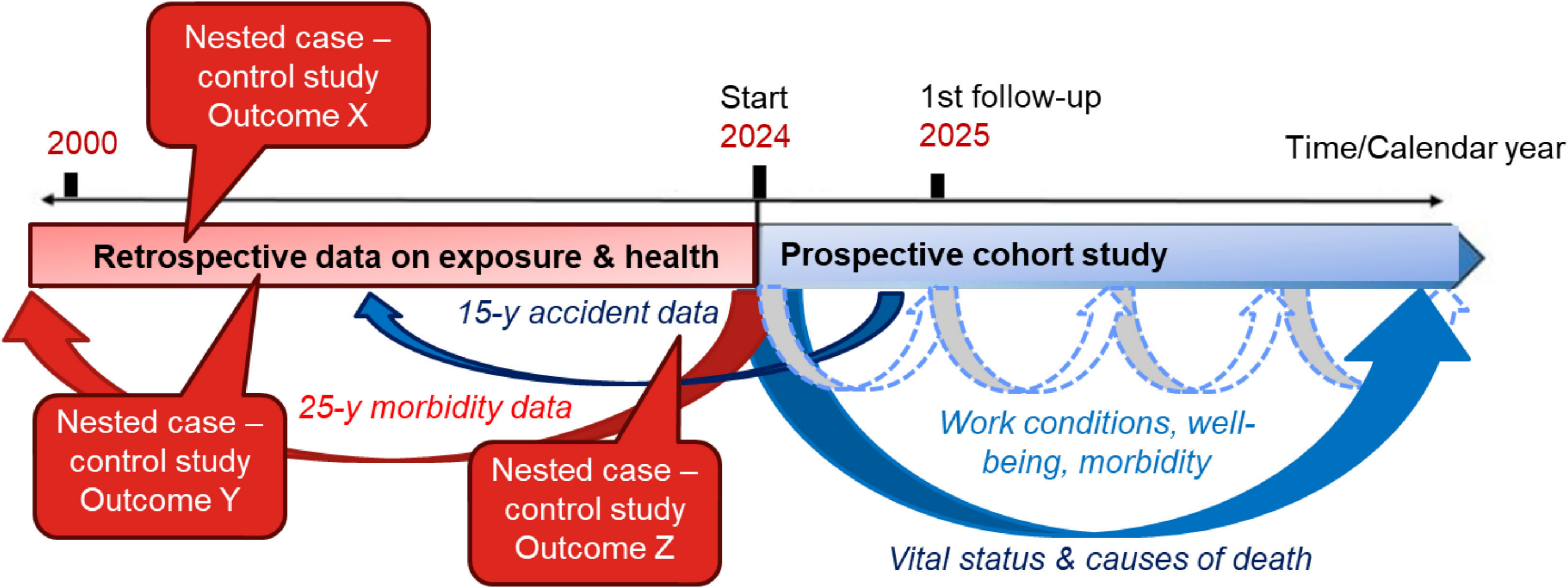
Design of the Swiss Transport Personnel Health Cohort study

Given above-mentioned considerations, we first considered an ambidirectional cohort design. Indeed, hospital data and cancer registries available 25 and 5 years backward along with the retrospective individual exposure assessment enables identifying cases starting from the latest between the date of the 1^st^ employment as bus drivers or the date of the registry opening. However, such a strategy could entail time zero bias [45] and/or immortal time bias [46], especially if selection effect is present [47]. Some outcomes and exposures are less susceptible to these biases (e.g. exposures that wouldn’t influence an employee to leave work or outcomes that are not associated with employment outside of exposure). For instance, outcomes such as obstructive sleep apnea, hearing loss, and hypertension which might be undiagnosed for a long time and chronic exposures such as air pollution, vibrations, noise could be studied using retrospective exposure. Otherwise, an unbiased way to exploit retrospective data would be using nested matched case-control studies focused on the outcome of interest (Fig. 1) and further emulating a target trial using records under a case-control sampling [48].

### Participant eligibility criteria

Swiss residents of both sexes employed as professional bus drivers for at least 1 year in a Swiss public transport company are eligible for participation. Cross-border workers (i.e., bus drivers working in Switzerland but living in neighboring country), former or retired bus drivers are non-eligible. In fact, data linkage with available Swiss registries is impossible for the former, while for the latter, an accurate assessment of bus history and work conditions would be challenging.

### Participant enrolment procedure

Three complementary channels are used in parallel for study advertising: 1-the unions who email the information to the unionized bus drivers and distribute advertising flyers in the companies under their influence; 2-the UTP who emails the study invitation package to the affiliated companies (90% of Swiss public transport companies) demanding them to distribute it to their bus drivers; and 3-the institutional social media. All information supports (flyers, emails, and posts) contain a link and a QR code of the study website (https://www.trapheac.ch/fr/soutenir-trapheac). Web-site visitors are asked whether they wish to participate in TRAPHEAC and those who agree are directed to the eligibility screening page. Confirmed eligible bus drivers are then directed to the registration page, where they must provide their identifying information, email and home address, read the study information letter and electronically signed the informed consent form. The information collected at this step is separated from all other data. Those who consented to participate receive by email the link to the inclusion questionnaire along with the login and the password.

### Inclusion questionnaire and data collection at baseline

The inclusion questionnaire aims at assessing all important data for etiological and interventional research. Whenever possible, we used the existing standardized and validated questions and instruments to enable national and international comparisons. Most questions on demographics, anthropometrics, lifestyle, women’ heath, medical history and treatments were borrowed from the pilot Swiss Health Study [39]. Exposure history and history of driven buses were assessed using questions developed and validated in the feasibility study [19]. Questions on bus drivers’ work and rest conditions were co-developed with unions. The questionnaire was translated in three national languages (Italian, French and German) and proofread and approved by drivers’ representatives (Additional file 1). An electronic three-lingual questionnaire was then developed on REDCap, tested online by team members and drivers’ representatives and open for self-administration upon individual invitation. The estimated time for questionnaire completion is 45–60 minutes.

Additional variables regarding household composition, accommodation characteristics and residential exposures at baseline will be extracted from the national registries and monitoring systems (Table 1) and linked with the participants’ data by the Swiss Statistical Office (FSO). Deterministic linkage procedure will be used for those who provided the social security number, probabilistic linkage based on individual registration data will be used otherwise. Similarly, the FSO will search back to 2000 hospital discharge records, extract diagnoses and treatment data and link them with participants.

### Follow-up questionnaire

The follow-up questionnaire aims at assessing changes in work and life conditions, exposures and health outcomes. We plan to distribute it every year and reduce the completion time to 15–20 minutes. Whenever possible, we will use the short versions of the PROMs, provided their validation in baseline data analysis.

### Data management and statistical analyses

Data will be extracted from REDcap, curated by the study data-manager using R statistical software, and stored according to the data-management plan validated by the Swissethics. All PROMs will be examined for structural and construct validity prior to statistical analyses. All statistical analysis will be based on the pre-established statistical analysis plans, precising the research question and hypotheses and statistical methods. After preliminary descriptive analyses, causal inference analysis is planned for assessing the causes of MSD and disentangling the joined and separate effects of occupational exposures, lifestyle-related and individual factors. Indeed, MSD constitute the most prevalent health problem in Swiss bus drivers [19] and will be given priority. The role of work environment, noise and EMF exposures on the sleep quality and well-being will be assessed as a second priority, at the main sponsor’s demand. We plan to consult bus drivers and their representatives to establish the list of priorities from their perspective and take it into account in the future analysis.

## Discussion

In this paper, we presented the development of the TRAPHEAC study protocol and described its mains components. The original design of this study is worth noting and will allow to conduct numerous studies nested into cohort and exploiting existing data. Hospital discharge data are, indeed, highly valuable medical data with precise diagnosis dates and treatment modalities accumulated for up to 25 years backward. Importantly, hospital discharge data contain nearly all diseases and disorders diagnosed during a hospital stay of at least one night. Not limiting the list of diseases to specific nosological categories is also a strength, as it allows research to be extended to all types of pathologies, including those that are rarely investigated. Bus driver studies seldom use diagnostic data for outcome assessment. In Switzerland, such data have never been used before, despite the FSO’s expertise in data linkage. These data will enable prevalence assessment for diagnosed diseases and disorders recorded at baseline and analyzed prospectively in incidence studies, or cross-sectionally at baseline. They will be regularly updated by the FSO in the future follow-ups.

The usage of BEM and BERM based on exposure measurement data is another important strength of this study. This enables retrospective individual exposure assessment annually and cumulatively since the start of at risk period, but also prevents the common method variance bias [49]. Via regular updating of the matrices by integrating new bus models it should be possible to update individual exposure estimates in the future follow-ups.

Notwithstanding, the TRAPHEAC protocol has also some limitations. TRAPHEAC is an observational study, and thus can be subject to bias. Some of them have been anticipated and prevented at the protocol development stage or could be controlled in statistical analysis, others would need a specific investigation. For example, random sample use was impossible as no national bus driver registry does exist in Switzerland. Using HR records of transport companies was deemed unsuitable by bus drivers and unions for confidentiality disclosure reasons. As we anticipated potential selection bias due to voluntary participation and potential self-selection of non-representative bus drivers, we introduced a 3-item non-respondent questionnaire to check the refusal reasons and quantify the bias magnitude [50]. We will also compare the study sample demographics with the national demographics of bus drivers (available at the FSO) and demographics of unionized bus drivers to examine how the selection might operate, if any, and reinforce the study promotion effort among subgroups difficult to reach.

One might also expect some information and classification bias that could be non-entirely prevented through the multisource data use. From our previous experience, we know that probabilistic data linkage could be less effective than deterministic one [17, 51, 52]. In the Swiss national cohort, unlinked records represented 6.9%, though most were related to individuals aged 10–29 years [53]. Although few Swiss bus drivers belong to this age group, this can lead to missing data on all external variables for unlinked drivers. As almost a half of the bus drivers (54%) were unwilling to provide their social security number in the feasibility study, information and classification bias will deserve precise quantification [47].

The bus history assessment is another point of discussion, as almost half of the participants were unable to complete it correctly in the feasibility study. Although we have simplified it in the inclusion questionnaire, it might still be challenging to complete, especially for those with long careers as bus drivers. The technical nature and length of the questionnaire—requiring recall of dates and details regarding work organization, bus models, and lifestyle—are critical. Both could lead to dropout and incomplete data. However, given the wide range of exposures and health outcomes among bus drivers, it was difficult to focus on only some of them or to disregard potential confounders, mediators, or moderators. This choice comes at the cost of participation and attrition rates, but providing compensation (we offer 25 Swiss francs per completed inclusion questionnaire) may help mitigate this limitation.

We believe that, overall, the study has a unique design and several methodologically strong aspects that will offset its potential limitations and generate robust new evidence on the causes and mechanisms of bus drivers’ ill health. This evidence is particularly timely for informing targeted preventive actions in this population, especially considering a growing bus driver shortage in nearly all countries. The yearly assessment of both exposure and health outcomes should enable capturing changes in work conditions and their effects on bus drivers’ health and well-being over time. Subsequently, TRAPHEAC could become an observatory of bus driver’s health at work and facilitate the implementation and evaluation of preventive interventions.

## Supplementary information

**Additional file 1:** Content of inclusion questionnaire administered at baseline on REDCap.

## Declarations

### Availability of data and material

Study material is available in a repository (https://doi.org/10.16909/dataset/59). There are no new data associated with this article.
