# Supplementary material for: Transport Personnel Health Cohort (TRAPHEAC): study protocol and methodological considerations
Source: Environ Health Prev Med. 2025 Jul 19;30:57. doi: 10.1265/ehpm.25-00127 (PMC12301074; doi:10.1265/ehpm.25-00127)
Supplement: Supplementary file 1 — Additional file 1: Content of inclusion questionnaire administered at baseline on REDCap. [file ehpm-30-057-s001.pdf]

Transport Personnel Health Cohort Study (TRAPHEAC)

Short Baseline Survey V2

IDENTIFYING AND GEOGRAPHICAL DATA

[IT Service]

A FEW QUESTIONS ABOUT YOU

In order to carry out the study, we would be grateful if you could provide some identification data. This information is essential to ensure the reliability of the analysis and to simplify your participation in the study.

The identification data, including your surname, first name, date of birth, address and ideally your AVS number, will be used by the Federal Statistical Office (FSO) to associate the data accurately and error-free. This approach aims to facilitate your participation by limiting the number and complexity of the questions asked.

Identification data will be stored and secured by Unisanté's IT department. More specifically, they will be stored and maintained with rigorous protection on a dedicated, secure server located in Switzerland and managed by authorized professionals for technical operations only. Only these people will have access to your data for IT management purposes (e.g. maintenance operations, portal management). The entire process is approved by the relevant ethics committee (CER-VD), guaranteeing compliance with ethical standards and the confidentiality of your information.

You will be assigned two personal, unique and de-identifying participant numbers. The first is for technical reasons and will be managed by Unisanté's IT department. The second will be the identifier used during the study. Only the SFO will have the correspondence between this study identifier and your identifying information.

We remind you that your answers will be treated as strictly confidential. The scientists carrying out the analyses will not have access to your identifying data. None of the individual results will be made public or communicated.

The questionnaire takes about 45-60 minutes to complete. We ask you to complete each section as completely as possible. You are free not to answer all the questions, but the value of the results of this survey depends above all on the information collected being as complete as possible.

1. What is/are your name(s)?

____

2. What is/are your first name(s)?

____

3. What is your date of birth?

☐☐ . ☐☐ . ☐☐☐☐

Day Month Year

4. What is your current address?[1] *(drop-down list)*

Path | N°

Additional address

Postal code | Location

Canton

Floor to ground :

-6+	-5	-4	-3	-2	-1	0	1	2	3	4	5	6	7	8	9	10+
☐	☐	☐	☐	☐	☐	☐	☐	☐	☐	☐	☐	☐	☐	☐	☐	☐

5. What is your AVS number? (optional)

☐☐☐ . ☐☐☐☐ . ☐☐☐☐☐☐

[1] We need this question to find out how much radon is present in your home's soil. Radon is a naturally occurring element in the earth, but its concentration can vary according to geographical region.

Demographics: Personal Information

A FEW QUESTIONS ABOUT YOU

This information is **essential** for understanding the survey results. The results **will not be individually examined**, but will be grouped together with the responses of all participants. This will enable us to make group analyses based on the criteria listed below.

1. What is your biological sex?

☐ Male ☐ Female ☐ Other

2. If Other: If you identify with another gender, please specify:

____

3. What is your current marital status?

☐ Single ☐ Married ☐ Divorced ☐ Widowed

☐ Unmarried - in a registered partnership

☐ Dissolved partnership (Judicially, by death or after declaration of absence)

4. What is your highest level of education? *(one answer only)*

☐ Primary/secondary school degree/diploma

☐ High school diploma

☐ CFC / Apprenticeship / Higher education professional maturity

☐ Higher education: Bachelor's

☐ Higher education: Master's degree/licence

☐ Higher education: Doctorate/PhD

5. Have you completed an apprenticeship?

☐ Yes ☐ No

5.1 If yes, in which field/sector of activity?

____

6. Did you have a professional activity before becoming a bus driver?

☐ Yes ☐ No

6.1 If yes, which one?

Profession / Occupation: _________

6.2 In which economic sector?

Business sector: ________

7. Are you still working as a bus driver?

☐ Yes ☐ No **=> remove following section: Working conditions**

7.1 What is your current situation (only one answer possible) :

☐ Retired

☐ Professional retraining

☐ On disability pension or permanent total work incapacity

☐ Looking for work

☐ Homemaker

☐ Another profession

8. If not, are you currently professionally active?

☐ Yes ☐ No → skip Part C, Q3

8.1 If yes. What is your current activity?

Professional activity: ________

8.2 In which business sector?

Sector of activity : ________

8.3 When did you stop working as a bus driver?

________ (DD:MM:YY)

Working conditions

We're now going to ask you a few questions about your working conditions. First, we'll ask you about your usual working hours, breaks and working environment. Secondly, we'll ask you how you currently experience these working conditions.

Clarification of the term "week": In this questionnaire, when we mention the term "week", we are referring to a block of work between two non-working days (your official rest days).

1. During the last 12 months, have you had irregular working hours? (Hours that change a lot during the month, such as working days and nights, or with very different shifts from one week to the next).

☐ Yes ☐ No→ **go to Q3**

1.1 If yes, how many times a month do you have to work irregular hours (on average, over the last 12 months)?

☐ All the time
☐ Three-quarters of the time
☐ Half the time
☐ A quarter of the time
☐ Occasionally

2. Do you have a schedule rotation?

☐ Yes, regular ☐ Yes, irregular ☐ No→ **go to Q4**

2.1 If yes to 2, How often are schedules rotated?

☐ Daily change ☐ Weekly change

☐ Monthly change ☐ Quarterly change

☐ Change every six months ☐ Other

2.2 If yes to 3, Do you have a change of tour of duty / rotation :

☐ **Cyclic** (e.g. morning, afternoon, evening, night, then repeat in the same order)

☐ **Alternate** (e.g. morning, afternoon, evening, night, then change order, e.g. evening, afternoon, morning)

☐ **Variable** (randomly changing schedules)

2.3 What is your work pattern in terms of consecutive days worked before one or more days off?

☐ **My rhythm is regular:**
I usually work ___ consecutive days before one or more days off.

☐ 1 day ☐ 2 days ☐ 3 days ☐ 4 days ☐ 5 days ☐ 6 days

☐ 7 days ☐ 8 days ☐ 9 days ☐ 10 days ☐ 11 days ☐ 12 days ☐ 13 days

☐ **My rhythm is irregular/variable:**
I sometimes work up to ___ consecutive days before one or more days off.

☐ 1 day ☐ 2 days ☐ 3 days ☐ 4 days ☐ 5 days ☐ 6 days

☐ 7 days ☐ 8 days ☐ 9 days ☐ 10 days ☐ 11 days ☐ 12 days ☐ 13 days

☐ **There's no pattern**

2.4 How many days off do you have between consecutive working days?

☐☐ days

<u>We now ask you to take a week as a block of work, which lies between two non-working days.</u>

3. On average, how often do you work between 04h and 06h per week (work block)? *(one answer possible)*

☐ 1 time a week ☐ 2 times a week
☐ 3 times a week ☐ 4 times a week
☐ 5 times a week ☐ 6 times a week
☐ 7 times a week

4. If yes to In the evening Q1. On average, how often do you work between 8 p.m. and midnight per week (work block)? *(one answer possible)*

☐ 1 time a week ☐ 2 times a week
☐ 3 times a week ☐ 4 times a week
☐ 5 times a week ☐ 6 times a week
☐ 7 times a week

5. If yes to At night Q1. On average, how often do you work between 00:00 and 04:00 per week (work block)? *(one answer possible)*

☐ 1 time a week ☐ 2 times a week
☐ 3 times a week ☐ 4 times a week
☐ 5 times a week ☐ 6 times a week
☐ 7 times a week

6. During the last 12 months, have you also worked nights (between 11pm and 6am)

☐ No
☐ Yes, occasionally (less than 3 times a week on average over the year)
☐ Yes, regularly (more than 3 times a week on average over the year)

7. How many hours a DAY do you usually work?

☐☐ hours AND ☐☐☐ min /day

8. How many hours per WEEK (work block) do you usually work?

☐☐☐ hours/week

9. How many times a week have you worked over 10 hours?

Amplitude refers to the time between the start and end of the working day, including breaks.

☐ Never ☐ 1 time a week ☐ 2 times a week
☐ 3 times a week ☐ 4 times a week
☐ 5 times a week ☐ 6 or more times a week
☐ 7 or more times a week

10. What days of the week do you usually work? *(Multiple answers possible)*

☐ Weekdays (Monday - Friday) ☐ Saturday
☐ Sunday

10.1 If yes for Saturday. Have you worked on any of the last 4 Saturdays?

☐ Yes ☐ No

10.2 If yes for Sunday. Have you worked on any of the last 4 Sundays?

☐ Yes ☐ No

11. During your working day, do you drive in pleasant environments?

☐ Yes ☐ No

11.1 If yes, why do you find them pleasant? *(one answer per line)*

- 11.1.1 Green spaces ☐ Yes ☐ No
- 11.1.2 Blue spaces (rivers, lakes) ☐ Yes ☐ No
- 11.1.3 Monuments, architecture ☐ Yes ☐ No
- 11.1.4 Bright, open spaces ☐ Yes ☐ No
- 11.1.5 Ease of driving ☐ Yes ☐ No

12. During your driving day, do you find yourself in traffic jams? *(one answer possible)*

☐ Never ☐ Rarely (1-2h a day)
☐ Frequently (2-5h a day) ☐ All the time

Your paid breaks and terminal stop

13. In general, how many paid breaks do you take a day?

☐☐☐ breaks/day

14. In general, how long does each break last?

☐☐☐ minutes

15. During these breaks, do you have access to:

	Never	Rarely	Sometimes	Often	Very often
**Toilets**	☐	☐	☐	☐	☐
**A break area**	☐	☐	☐	☐	☐
**Water**	☐	☐	☐	☐	☐
**A coffee or beverage dispenser**	☐	☐	☐	☐	☐

15.1 If rarely, sometimes, often or very often for the break area. Describe this break area *(several answers possible)*

Outdoors

- ☐ A beautiful natural landscape
- ☐ Attractive cityscape
- ☐ A pretty rural landscape
- ☐ The presence of plants or flowers
- ☐ Is airy
- ☐ Nothing remarkable

Inside

- ☐ Green plants
- ☐ Is calm
- ☐ Includes games for relaxing (e.g. table soccer)
- ☐ Sports equipment
- ☐ Armchair, sofa, relaxation area
- ☐ Nothing remarkable

16. On a normal working day, how much time can you spend in a break area?

☐☐☐ breaks/day

17. In general, how many times a day do you stop at the terminus?

☐☐☐ minutes

18. In general, how long does a stop at the terminus last?

☐☐☐ minute(s)

19. During your terminal stops, do you have access to : *(one answer per line)*

	Never	Rarely	Sometimes	Often	Very often
**Toilets**	☐	☐	☐	☐	☐
**A break area**	☐	☐	☐	☐	☐
**A water**	☐	☐	☐	☐	☐
**A coffee or beverage dispenser**	☐	☐	☐	☐	☐

19.1 If rarely, sometimes, often or very often for the break area. Describe this break area *(several answers possible)*

Outdoors

- ☐ A beautiful natural landscape
- ☐ Attractive cityscape
- ☐ Beautiful rural scenery
- ☐ Presence of plants or flowers
- ☐ Is airy
- ☐ Nothing remarkable

Inside

- ☐ Green plants
- ☐ Is calm, unhurried
- ☐ Games for relaxing (e.g. table soccer)
- ☐ Sports equipment
- ☐ Armchair, sofa, relaxation area
- ☐ Nothing remarkable

Scale: BORG → Perception of effort

20. How would you rate the intensity of the physical effort involved in your work during a typical working day? Tick the number corresponding to your choice on the scale from 0 to 10 below, ranging from "No effort" to "Maximal effort": *(one answer possible)*

No effort	Very light	Light	Moderate	Somewhat hard	Hard	Very hard	Extremely hard	Near maximum effort	Maximal effort
0	0.5	1	2	3	4	5	6	7-9	10
☐	☐	☐	☐	☐	☐	☐	☐	☐	☐

Scale: Work-Family Conflict

21. We are now going to ask you some questions about your feelings at work. Please choose to what extent you agree or disagree with the sentences below. *(one answer per line)*

	Strongly disagree	Disagree	Slightly disagree	Neither agree nor disagree	Slightly agree	Agree	Strongly Agree
**The demands of my work interfere with my home and family life.**	☐	☐	☐	☐	☐	☐	☐
**The amount of time my job takes up makes it difficult to fulfill family responsibilities.**	☐	☐	☐	☐	☐	☐	☐
**Things I want to do at home do not get done because of the demands my job puts on me.**	☐	☐	☐	☐	☐	☐	☐
**My job produces strain that makes it difficult to fulfill family duties.**	☐	☐	☐	☐	☐	☐	☐
**Due to work-related duties, I have to make changes to my plans for family activities.**	☐	☐	☐	☐	☐	☐	☐

Scale: Effort-Reward Imbalance Questionnaire - Short (ERI-S)

22. The following items relate to your current profession. Indicate whether you agree or disagree with each sentence by ticking the corresponding box *(one answer per line).*

	Strongly disagree	Disagree	Agree	Strongly Agree
I have constant time pressure due to a heavy workload.	☐	☐	☐	☐
I have many interruptions and disturbances while performing my job.	☐	☐	☐	☐
Over the past few years, my job has become more and more demanding.	☐	☐	☐	☐
I receive the respect I deserve from my superior or a respective relevant person.	☐	☐	☐	☐
My job promotion perspectives are poor.	☐	☐	☐	☐
I have experienced or I expect to experience an undesirable change in my work situation.	☐	☐	☐	☐
My job security is poor.	☐	☐	☐	☐
Considering all my efforts and achievements, I receive the respect and prestige I deserve at work.	☐	☐	☐	☐
Considering all my efforts and achievements, my job promotion prospects are adequate.	☐	☐	☐	☐
Considering all my efforts and achievements, my salary / income is adequate.	☐	☐	☐	☐
I get easily overwhelmed by time pressures at work.	☐	☐	☐	☐
As soon as I get up in the morning I start thinking about work problems.	☐	☐	☐	☐

When I get home, I can easily relax and 'switch off' work.	☐	☐	☐	☐
People close to me say I sacrifice too much for my job.	☐	☐	☐	☐
Work rarely lets me go, it is still on my mind when I go to bed.	☐	☐	☐	☐
If I postpone something that I was supposed to do today I'll have trouble sleeping at night.	☐	☐	☐	☐

Scale: 1-item measure of job stressfulness

23. In general, how do you find your job? *(one answer possible).*

Not at all stressful	**Mildly stressful**	**Moderately stressful**	**Very stressful**	**Extremely stressful**
☐	☐	☐	☐	☐

Scale: Perceived Occupational Stress Scale

24. Rate the extent to which you agree or disagree with the following propositions: *(one answer per line)*

	**Strongly disagree**	**Disagree**	**Neither agree nor disagree**	**Agree**	**Strongly Agree**
**My work is stressful**	☐	☐	☐	☐	☐
**Thinking about my work makes me feel tense**	☐	☐	☐	☐	☐
**At work I feel under pressure**	☐	☐	☐	☐	☐

**My work has negative effects on my health**	☐	☐	☐	☐	☐

Scale: Burnout Assessment Tool - Short (BAT-S)

25. The following statements relate to how you feel about your work and how you experience it. Please indicate how **often** each statement applies to you. (*One answer per line)*

	Never	Rarely	Sometimes	Often	Always
**1. At work, I feel mentally exhausted**	☐	☐	☐	☐	☐
**2. After a day at work, I find it hard to recover my energy**	☐	☐	☐	☐	☐
**3. At work, I feel physically exhausted**	☐	☐	☐	☐	☐
**4. I struggle to find any enthusiasm for my work**	☐	☐	☐	☐	☐
**5. I feel a strong aversion towards my job**	☐	☐	☐	☐	☐
**6. I'm cynical about what my work means to others**	☐	☐	☐	☐	☐
**7. At work, I have trouble staying focused**	☐	☐	☐	☐	☐
**8. I'm forgetful and distracted at work**	☐	☐	☐	☐	☐
**9. I make mistakes in my work because I have my mind on other things**	☐	☐	☐	☐	☐
**10. At work, I feel unable to control my emotions**	☐	☐	☐	☐	☐
**11. I do not recognize myself in the way I react emotionally at work**	☐	☐	☐	☐	☐
**12. At work I may overreact unintentionally**	☐	☐	☐	☐	☐

26. Please indicate how often you encounter the following situations in your current job. *(one answer per line)*

	Less than once a month or never	1 time per month	2-3 times a month	About once a week	2-3 times a week approx.	Approx. once a day	Several times a day
**Outside the bus...**							
I suffer from the aggression of other road users (cars, bikes).	☐	☐	☐	☐	☐	☐	☐
I'm afraid of having an accident with another vehicle (car, truck).	☐	☐	☐	☐	☐	☐	☐
I'm afraid of having an accident with a bicycle, scooter or motorcycle.	☐	☐	☐	☐	☐	☐	☐
I'm afraid of having an accident with my vehicle	☐	☐	☐	☐	☐	☐	☐
I'm afraid of causing an accident	☐	☐	☐	☐	☐	☐	☐
**Inside the bus...**							
I'm subjected to verbal aggression from bus passengers	☐	☐	☐	☐	☐	☐	☐
I'm afraid of having an accident on my bus, causing injuries.	☐	☐	☐	☐	☐	☐	☐

27. In the last 12 months, at your work, have you been subject to... *(several answers possible)*

- ☐ Age discrimination
- ☐ Discrimination based on your sex or gender
- ☐ Discrimination based on your nationality, ethnic origin or skin color
- ☐ Verbal abuse
- ☐ Threats and humiliating behaviour
- ☐ Physical violence

- ☐ Bullying, harassment, mobbing
- ☐ Sexual harassment
- ☐ None of this discrimination or violence

Your sleep→ Scale: GSAQ

1. We're going to ask you a few questions about your <u>sleep</u>. Choose the answer that best suits you.

	Never	Sometimes	Usually	Always
**Did you have difficulty falling asleep, staying asleep, or feeling poorly rested in the morning?**	☐	☐	☐	☐
**Did you fall asleep unintentionally or have to fight to stay awake during the day?**	☐	☐	☐	☐
**Did sleep difficulties or daytime sleepiness interfere with your daily activities?**	☐	☐	☐	☐
**Did work or other activities prevent you from getting enough sleep?**	☐	☐	☐	☐
**Did you snore loudly?**	☐	☐	☐	☐
**Did you hold your breath, have breathing pauses, or stop breathing in your sleep?**	☐	☐	☐	☐
**Did you have restless or "crawling" feelings in your legs at night that went away if you moved your legs?**	☐	☐	☐	☐
**Did you have repeated rhythmic leg jerks or leg twitches during your sleep?**	☐	☐	☐	☐
**Did you have nightmares, or did you scream, walk, punch, or kick in your sleep?**	☐	☐	☐	☐
**Did the following things disturb your sleep:**				
**a. Pain**<br>**Please specify : ________**	☐	☐	☐	☐

**b. Other physical problems**<br>**Please specify : _________**	☐	☐	☐	☐
**c. Worries**<br>**Please specify : _________**	☐	☐	☐	☐
**d. Medications**<br>**Please specify : _________**	☐	☐	☐	☐
**e. Other**<br>**Please specify : _________**	☐	☐	☐	☐
**Did you feel sad or anxious?**	☐	☐	☐	☐

YOUR HEALTH

We will now ask you a few questions about your current state of health and your past history. Please answer each question as accurately as possible. We remind you that your answers will remain anonymous and will be analyzed together with those of your colleagues, without individual identification.

A few questions about your health

1. What is your weight?

☐☐☐ kg

2. What is your current height?

☐☐☐ cm

3. Are you right- or left-handed?

Please tick only one answer.

☐ Right-handed ☐ Left-handed ☐ Both

4. On a scale of 1 to 10, how would you rate your general state of health (both physical and mental)?

1- being very bad, 10- being very good

☐☐

Musculoskeletal disorders→ Modified Nordic Musculoskeletal Questionnaire

If you are not currently working, please think about your daily activities instead of work.

Explanation:

The health questions cover the following 9 body parts.

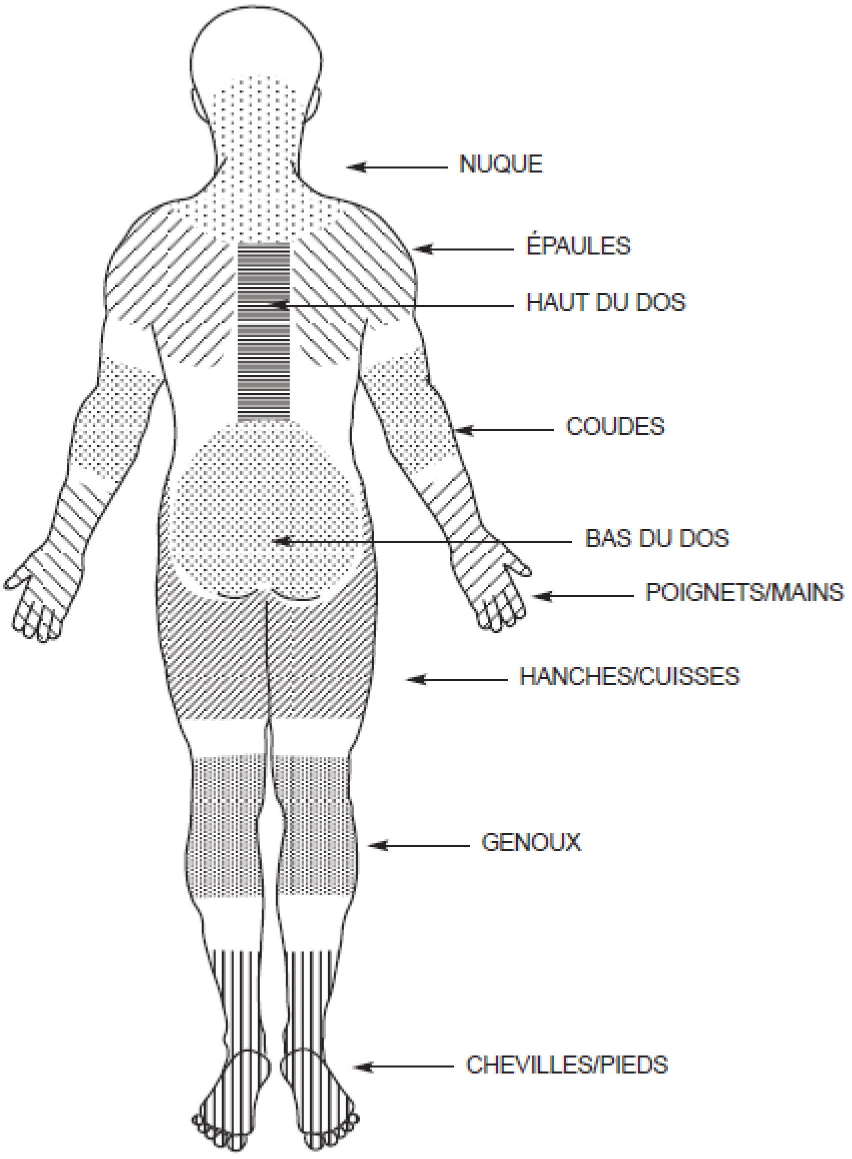

We ask you to answer the questions for each body part, even if you do not feel any pain or symptoms in that part of the body.

1. Neck

1. <u>Over the past 12 months</u>, how much pain have you experienced in your neck? *Please tick one answer only*

**Neck**	**No pain**	☐ **0**	☐ **1**	☐ **2**	☐ **3**	☐ **4**	☐ **5**	☐ **6**	☐ **7**	☐ **8**	☐ **9**	☐ **10**	**Worst pain ever**

If 0, go to part 2. Shoulder

If >0, answer questions 1.1 to 1.6

1.1 <u>Over the past 12 months</u>, what is the <u>total</u> length of time that you have suffered from neck pain? *Please tick one answer only*

	Less than 24 hours	From 1 to 7 days	8 to 30 days	More than 30 days	All the time
**Neck**	☐	☐	☐	☐	☐

1.2 To what extent has this neck pain limited your ability to work? *Please tick one answer only*

**Neck**	**No limitation**	☐ **0**	☐ **1**	☐ **2**	☐ **3**	☐ **4**	☐ **5**	☐ **6**	☐ **7**	☐ **8**	☐ **9**	☐ **10**	**Inability to work due to neck pain**

1.3 <u>In the last 12 months</u>, have you ever had to stop work because of this neck pain? *Please tick one answer only*

☐ Yes ☐ No

1.4 <u>In the last 12 months</u>, have you ever had to change/adapt your job or workstation because of neck pain? *Please tick one answer only*

☐ Yes ☐ No

2. Shoulders

2. <u>Over the past 12 months</u>, how much pain have you experienced in either shoulder? *Please tick one answer only*

**Shoulder**	**No pain**	☐ **0**	☐ **1**	☐ **2**	☐ **3**	☐ **4**	☐ **5**	☐ **6**	☐ **7**	☐ **8**	☐ **9**	☐ **10**	**Worst pain ever**

If 0, go to part 3. Upper back

If >0, answer questions 2.1 to 2.6

2.1 <u>Over the past 12 months</u>, what is the <u>total</u> length of time that you have suffered from shoulder pain? *Please tick one answer only*

	Less than 24 hours	From 1 to 7 days	8 to 30 days	More than 30 days	All the time
**Shoulder**	☐	☐	☐	☐	☐

2.2 To what extent has this shoulder pain limited your ability to work? *Please tick one answer only*

**Shoulder**	**No limitation**	☐ **0**	☐ **1**	☐ **2**	☐ **3**	☐ **4**	☐ **5**	☐ **6**	☐ **7**	☐ **8**	☐ **9**	☐ **10**	**Inability to work due to shoulder pain**

2.3 <u>In the last 12 months</u>, have you ever been off work due to shoulder pain? *Please tick one answer only*

☐ Yes ☐ No

2.4 <u>In the last 12 months</u>, have you ever had to change/adapt your job or workstation because of shoulder pain? *Please tick one answer only*

☐ Yes ☐ No

3. Upper back

3. <u>Over the past 12 months</u>, how much pain have you experienced in your upper back? *Please tick one answer only*

**Upper back**	**No pain**	☐ **0**	☐ **1**	☐ **2**	☐ **3**	☐ **4**	☐ **5**	☐ **6**	☐ **7**	☐ **8**	☐ **9**	☐ **10**	**Worst pain ever**

If 0, go to part 4. Elbow

If >0, answer questions 3.1 to 3.6

3.1 <u>Over the last 12 months</u>, what is the <u>total</u> length of time that you have suffered from upper back pain? *Please tick one answer only*

	Less than 24 hours	From 1 to 7 days	8 to 30 days	More than 30 days	All the time
**Upper back**	☐	☐	☐	☐	☐

3.2 To what extent has this upper back pain limited your ability to work? *Please tick one answer only*

Upper back	**No limitation**	☐ 0	☐ 1	☐ 2	☐ 3	☐ 4	☐ 5	☐ 6	☐ 7	☐ 8	☐ 9	☐ 10	**Inability to work due to upper back pain**

3.3 <u>In the last 12 months</u>, have you ever had to stop work because of this pain in your upper back? *Please tick one answer only*

☐ Yes ☐ No

3.4 <u>In the last 12 months</u>, have you ever had to change/adapt your job or workstation because of upper back pain? *Please tick one answer only*

☐ Yes ☐ No

4. Elbow

4. <u>Over the past 12 months</u>, how much pain have you experienced in one or both elbows? *Please tick one answer only*

**Elbow**	**No pain**	☐ **0**	☐ **1**	☐ **2**	☐ **3**	☐ **4**	☐ **5**	☐ **6**	☐ **7**	☐ **8**	☐ **9**	☐ **10**	**Worst pain ever**

If 0, go to part 5. Lower back

If >0, answer questions 4.1 to 4.6

4.1 <u>Over the past 12 months</u>, what is the <u>total</u> length of time that you have suffered from pain in one or both elbows? *Please tick one answer only*

	Less than 24 hours	From 1 to 7 days	8 to 30 days	More than 30 days	All the time
**Elbow**	☐	☐	☐	☐	☐

4.2 To what extent has this pain in one or both elbows limited your ability to work? *Please tick one answer only*

**Elbow**	**No limitation**	☐ **0**	☐ **1**	☐ **2**	☐ **3**	☐ **4**	☐ **5**	☐ **6**	☐ **7**	☐ **8**	☐ **9**	☐ **10**	**Inability to work due to elbow pain**

4.3 <u>In the last 12 months</u>, have you ever had to stop work because of pain in one or both elbows? *Please tick one answer only*

☐ Yes ☐ No

4.4 <u>In the last 12 months</u>, have you ever had to change/adapt your job or workstation because of pain in one or both elbows? *Please tick one answer only*

☐ Yes ☐ No

5. Lower back

5. <u>Over the past 12 months</u>, how much pain have you experienced in your lower back? *Please tick one answer only*

**Lower back**	**No pain**	☐ **0**	☐ **1**	☐ **2**	☐ **3**	☐ **4**	☐ **5**	☐ **6**	☐ **7**	☐ **8**	☐ **9**	☐ **10**	**Worst pain ever**

If 0, go to part 6. Wrist/hand

If >0, answer questions 5.1 to 5.7

5.1 <u>Over the last 12 months</u>, what is the <u>total</u> length of time that you have suffered from lower back pain? *Please tick one answer only*

	**Less than 24 hours**	**From 1 to 7 days**	**8 to 30 days**	**More than 30 days**	**All the time**
**Lower back**	☐	☐	☐	☐	☐

5.2 To what extent has this lower back pain limited your ability to work? *Please tick one answer only*

Lower back	**No limitation**	☐ 0	☐ 1	☐ 2	☐ 3	☐ 4	☐ 5	☐ 6	☐ 7	☐ 8	☐ 9	☐ 10	**Inability to work due to lower back pain**

5.3 <u>In the last 12 months</u>, have you ever been off work due to this lower back pain? *Please tick one answer only*

☐ Yes ☐ No

5.4 <u>In the last 12 months</u>, have you ever had to change/adapt your job or workstation because of lower back pain? *Please tick one answer only*

☐ Yes ☐ No

6. Wrist/hand

6. <u>Over the last 12 months</u>, how much pain have you experienced in one or both wrists or hands?
Please tick one answer only

Wrist /hand	No pain	☐ 0	☐ 1	☐ 2	☐ 3	☐ 4	☐ 5	☐ 6	☐ 7	☐ 8	☐ 9	☐ 10	Worst pain ever

If 0, go to part 7. Hip/thigh
If >0, answer questions 6.1 to 6.6

6.1 In <u>the last 12 months</u>, what is the <u>total</u> length of time that you have suffered in one or both wrists or hands? *Please tick one answer only*

	Less than 24 hours	From 1 to 7 days	8 to 30 days	More than 30 days	All the time
**Wrist/hand**	☐	☐	☐	☐	☐

6.2 To what extent has this pain in one or both wrists or hands limited your ability to work? *Please tick one answer only*

**Wrist/hand**	**No limitation**	☐ **0**	☐ **1**	☐ **2**	☐ **3**	☐ **4**	☐ **5**	☐ **6**	☐ **7**	☐ **8**	☐ **9**	☐ **10**	**Inability to work due to wrist/hand pain**

6.3 <u>In the last 12 months</u>, have you ever had to stop work because of pain in **one or both wrists or hands?** *Please tick one answer only*

☐ Yes ☐ No

6.4 <u>In the last 12 months</u>, have you ever had to change/adapt your job or workstation because of pain in one or both wrists or hands? *Please tick one answer only*

☐ Yes ☐ No

7. Hip/thigh

7. <u>Over the past 12 months</u>, how much pain have you experienced in one or both hips/thighs?
Please tick one answer only

**Hip/thigh**	**No pain**	☐ **0**	☐ **1**	☐ **2**	☐ **3**	☐ **4**	☐ **5**	☐ **6**	☐ **7**	☐ **8**	☐ **9**	☐ **10**	**Worst pain ever**

If 0, go to part 8. Knee
If >0, answer questions 7.1 to 7.6

7.1 <u>Over the past 12 months</u>, what is the <u>total</u> length of time that you have suffered from pain in one or both hips/thighs? *Please tick one answer only*

	Less than 24 hours	From 1 to 7 days	8 to 30 days	More than 30 days	All the time
**Hip/thigh**	☐	☐	☐	☐	☐

7.2 To what extent has this pain in one or both hips/thighs limited your ability to work? *Please tick one answer only*

**Hip/thigh**	**No limitation**	☐ **0**	☐ **1**	☐ **2**	☐ **3**	☐ **4**	☐ **5**	☐ **6**	☐ **7**	☐ **8**	☐ **9**	☐ **10**	**Inability to work due to hip/thigh pain**

7.3 <u>In the last 12 months</u>, have you ever had to stop work because of this pain in one or both hips/thighs? *Please tick one answer only*

☐ Yes ☐ No

7.4 <u>In the last 12 months</u>, have you ever had to change/adapt your job or workstation because of pain in one or both hips/thighs? *Please tick one answer only*

☐ Yes ☐ No

8. Knee

8. <u>Over the past 12 months</u>, how much pain have you experienced in one or both knees? *Please tick one answer only*

Knee	No pain	☐ 0	☐ 1	☐ 2	☐ 3	☐ 4	☐ 5	☐ 6	☐ 7	☐ 8	☐ 9	☐ 10	Worst pain ever

If 0, go to part 9. Ankle and Foot

If >0, answer questions 8.1 to 8.6

8.1 <u>Over the past 12 months</u>, what is the <u>total</u> length of time that you have suffered from one or both knees? *Please tick one answer only*

	Less than 24 hours	From 1 to 7 days	8 to 30 days	More than 30 days	All the time
**Knee**	☐	☐	☐	☐	☐

8.2 To what extent has this pain in one or both knees limited your ability to work? *Please tick one answer only*

**Knee**	**No limitation**	☐ **0**	☐ **1**	☐ **2**	☐ **3**	☐ **4**	☐ **5**	☐ **6**	☐ **7**	☐ **8**	☐ **9**	☐ **10**	**Inability to work due to knee pain**

8.3 <u>In the last 12 months</u>, have you ever had to stop work because of pain in one or both knees? *Please tick one answer only*

☐ Yes ☐ No

8.4 <u>In the last 12 months</u>, have you ever had to change/adapt your job or workstation because of pain in one or both knees? *Please tick one answer only*

☐ Yes ☐ No

9. Ankle/foot

9. <u>Over the past 12 months</u>, how much pain have you experienced in one or both feet or ankles? *Please tick one answer only*

Ankle/foot	No pain	☐ 0	☐ 1	☐ 2	☐ 3	☐ 4	☐ 5	☐ 6	☐ 7	☐ 8	☐ 9	☐ 10	Worst pain ever

If 0, go to next section.

If >0, answer questions 9.1 to 9.6

9.1 <u>Over the past 12 months</u>, what is the <u>total</u> length of time that you have suffered from one or both feet or ankles? *Please tick one answer only*

	Less than 24 hours	From 1 to 7 days	8 to 30 days	More than 30 days	All the time
**Ankle/foot**	☐	☐	☐	☐	☐

9.2 To what extent has this pain in one or both feet or ankles limited your ability to work? *Please tick one answer only*

**Ankle/foot**	**No limitation**	☐ **0**	☐ **1**	☐ **2**	☐ **3**	☐ **4**	☐ **5**	☐ **6**	☐ **7**	☐ **8**	☐ **9**	☐ **10**	**Inability to work due to ankle/foot pain**

9.3 <u>In the last 12 months</u>, have you ever had to stop work because of pain in one or both feet or ankles? *Please tick one answer only*

☐ Yes ☐ No

9.4 <u>In the last 12 months</u>, have you ever had to change/adapt your job or workstation because of pain in one or both feet or ankles? *Please tick one answer only*

☐ Yes ☐ No

Well-being

Please indicate for each of the five statements which is closest to how you have been feeling over the last two weeks. Notice that higher numbers mean better well-being.

Example. If you have felt cheerful and in good spirits more than half of the time during the last two weeks, select number three.

In the last two weeks (*One answer possible per line)*

	**All of the time** 5	**Most of the time** 4	**More than half of the time** 3	**Less than half of the time** 2	**Some of the time** 1	**At no time** 0
**I have felt cheerful in good spirits**	☐	☐	☐	☐	☐	☐
**I have felt calm and relaxed**	☐	☐	☐	☐	☐	☐
**I have felt active and vigorous**	☐	☐	☐	☐	☐	☐
**I woke up feeling fresh and rested**	☐	☐	☐	☐	☐	☐
**My daily life has been filled with things that interest me.**	☐	☐	☐	☐	☐	☐

YOUR LIFE BALANCE

We'll now ask you a few questions about your life balance. There will be questions about your well-being, your eating and exercise habits, and so on. Please answer each question as precisely as possible. Please take your time to answer them.

Some of the questions we're about to ask you are sensitive. They may be more difficult to answer. If you feel uncomfortable answering these questions, don't hesitate to contact an association offering support by calling 143, or by writing to www.143.ch. This contact is completely anonymous.

Personality→ Scale: BFI-2-XS

1. Here are a number of characteristics that may or may not apply to you. For example, do you agree that you are someone who likes to spend time with others? Please write a number next to each statement to indicate the extent to which you agree or disagree with that statement. *(One possible answer)*

	Disagree strongly	Disagree a little	Neutral, no opinion	Agree a little	Agree strongly
Tends to be quiet	☐	☐	☐	☐	☐
Is compassionate, has a soft heart	☐	☐	☐	☐	☐
Tends to be disorganized	☐	☐	☐	☐	☐
Worries a lot	☐	☐	☐	☐	☐
Is fascinated by art, music, or literature	☐	☐	☐	☐	☐
Is dominant, acts as a leader	☐	☐	☐	☐	☐
Is sometimes rude to others	☐	☐	☐	☐	☐
Has difficulty getting started on tasks	☐	☐	☐	☐	☐
Tends to feel depressed, blue	☐	☐	☐	☐	☐
Has little interest in abstract ideas	☐	☐	☐	☐	☐

**Is full of energy**	☐	☐	☐	☐	☐
**Assumes the best about people**	☐	☐	☐	☐	☐
**Is reliable, can always be counted on**	☐	☐	☐	☐	☐
**Is emotionally stable, not easily upset**	☐	☐	☐	☐	☐
**Is original, comes up with new ideas**	☐	☐	☐	☐	☐

Quality of life and satisfaction→ WHOQOL-Bref scale

1. **Choose the statements that best describe how you feel** (*one possible answer per line).*

	Very poor	Poor	Neither poor nor good	Good	Very good
**How would you rate your quality of life?**	☐	☐	☐	☐	☐
	**Very dissatisfied**	**Dissatisfied**	**Neither satisfied nor dissatisfied**	**Satisfied**	**Very satisfied**
**How satisfied are you with your health?**	☐	☐	☐	☐	☐
	**Not at all**	**A little**	**A moderate amount**	**Very much**	**An extreme amount**
**To what extent do you feel that physical pain prevents you from doing what you need to do?**	☐	☐	☐	☐	☐
**How much do you need any medical treatment to function in your daily life?**	☐	☐	☐	☐	☐
**How much do you enjoy life?**	☐	☐	☐	☐	☐
**To what extent do you feel your life to be meaningful?**	☐	☐	☐	☐	☐
	**Not at all**	**A little**	**Moderately**	**Mostly**	**Completely**
**How well are you able to concentrate?**	☐	☐	☐	☐	☐

How safe do you feel in your daily life?	☐	☐	☐	☐	☐
How healthy is your physical environment?	☐	☐	☐	☐	☐
	Not at all	A little	Moderately	Mostly	Completely
Do you have enough energy for everyday life?	☐	☐	☐	☐	☐
Are you able to accept your bodily appearance?	☐	☐	☐	☐	☐
Have you enough money to meet your needs?	☐	☐	☐	☐	☐
How available to you is the information that you need in your day-to-day life?	☐	☐	☐	☐	☐
To what extent do you have the opportunity for leisure activities?	☐	☐	☐	☐	☐
	Very poor	Poor	Neither poor nor good	Well	Very well
How well are you able to get around?	☐	☐	☐	☐	☐
	Very dissatisfied	Dissatisfied	Neither satisfied nor dissatisfied	Satisfied	Very satisfied
How satisfied are you with your sleep?	☐	☐	☐	☐	☐
How satisfied are you with your ability to perform your daily living activities?	☐	☐	☐	☐	☐

How satisfied are you with your capacity for work?	☐	☐	☐	☐	☐
How satisfied are you with yourself?	☐	☐	☐	☐	☐
How satisfied are you with your personal relationships?	☐	☐	☐	☐	☐
How satisfied are you with your intimate life?	☐	☐	☐	☐	☐
How satisfied are you with the support you get from your friends?	☐	☐	☐	☐	☐
How satisfied are you with the conditions of your living place?	☐	☐	☐	☐	☐
How satisfied are you with your access to health services?	☐	☐	☐	☐	☐
How satisfied are you with your mode of transportation?					
	**Never**	**Seldom**	**Quite Often**	**Very often**	**Always**
How often do you have negative feelings, such as blue mood, despair, anxiety, depression?	☐	☐	☐	☐	☐

Nutrition→ Swiss Health Study (SHeS)

1. How often do you eat the following foods?

Two answers per line: food frequency and percentage of organic products.

									How often do you eat these organic products?			
	Rarely/never	1 time per month	Every 2 weeks	1-2 times a week	3-6 times a week	Every day	2-3 times a day	More than 4 times a day	<10%	10-50%	50-90%	>90%
Meat (beef, veal, pork, game, etc.), excluding poultry	☐	☐	☐	☐	☐	☐	☐	☐	☐	☐	☐	☐
Charcuterie (sausage, pâté, dried meat, etc.)	☐	☐	☐	☐	☐	☐	☐	☐	☐	☐	☐	☐
Wholegrain cereals (wholemeal bread, wholegrain rice, etc.)	☐	☐	☐	☐	☐	☐	☐	☐	☐	☐	☐	☐
Fruits	☐	☐	☐	☐	☐	☐	☐	☐	☐	☐	☐	☐
Sweets or desserts (chocolate, cookies, pastries, ice cream, cake, etc.)	☐	☐	☐	☐	☐	☐	☐	☐	☐	☐	☐	☐

2. Do you usually add salt to your food at the table?

	When you eat at home	When you eat out
No, never (0 meals out of 10)	☐	☐
Yes, occasionally (1 to 5 meals out of 10)	☐	☐
Yes, often (6 to 9 meals out of 10)	☐	☐
Yes, always (10 out of 10 meals)	☐	☐

3. How much of the following do you usually drink a day? 1 glass corresponds to 0.2 L. *One answer per line.*

	More than 3 liters per day	2-3 liters per day	1-2 liters per day	0.5- 1 liters per day	2-3 glasses per day (2-5 dL)	1 glass per day	1-6 glasses by week	Less than one glass by week
**Fruit juice (100% fruit juice)**	☐	☐	☐	☐	☐	☐	☐	☐
**Fruit nectar**	☐	☐	☐	☐	☐	☐	☐	☐
**Sweet tea, cola, lemonade, cold tea, syrup, chocolate drinks, energy drinks, etc. (excluding sweetened drinks, e.g. "light" or "zero").**	☐	☐	☐	☐	☐	☐	☐	☐
**Sweetened drinks (Diet Coke, Coke Zero, blue rivella, diet energy drinks, etc.)**	☐	☐	☐	☐	☐	☐	☐	☐
**Coffee**	☐	☐	☐	☐	☐	☐	☐	☐
**Unsweetened tea**	☐	☐	☐	☐	☐	☐	☐	☐
**Water (tap or mineral)**	☐	☐	☐	☐	☐	☐	☐	☐

Alcohol, Tobacco, Drugs

Alcohol

4. Has there ever been a time in your life when you consumed more than 5 glasses of alcoholic beverage (all beverages combined) every day?

☐ Yes ☐ No ☐ I don't know

5. Do you currently drink alcohol (alcoholic beverages)?

☐ Yes ☐ No ➔ **go to question 8 (tobacco and caffeine)**

6. How many bottles (3 dl) of beer have you drunk in the last 7 days?

☐☐ Bottles (0 if none)

7. How many glasses of wine (1 dl) have you drunk in the last 7 days?

☐☐ Glasses (0 if none)

8. How many glasses of spirits (cognac, kirsch, liqueur, whisky, brandy, etc.) (0.4 dl) have you drunk in the last 7 days?

☐☐ Glasses (0 if none)

Tobacco

9. Check the box that best describes you:

☐ I currently smoke ➔ **go to question 10**
☐ I used to smoke ➔ **go to question 11**
☐ I've never smoked ➔ **go to question 12**

10. What is the main product you currently smoke? (indicate quantity)

Cigarettes ☐☐ per day
Cigars ☐☐ per day (zero if less than 1/day)
Cigarillos ☐☐ per day (zero if less than 1/day)
Pipes ☐☐ per day (zero if less than 1/day)
E-cigarette ☐ liquid **with** ☐ and/or **without** ☐ nicotine?

11. At what age did you start/ stop smoking?

I started at __________ I stopped at __________

11a. During this period, for how many years did you stop, including all breaks?

__________ years

Passive smoking

12. In the last 12 months, were you exposed to tobacco smoke in your work environment (bus booths, breaks, etc.)?

☐ No, never ☐ Yes, sometimes ☐ Yes, regularly

13. In the last 12 months, were you exposed to tobacco smoke in your personal environment?

☐ No, never ☐ Yes, sometimes ☐ Yes, regularly

Drugs

14. Have you ever used drugs in your life, or do you currently use drugs (e.g. cannabis, cocaine...)?

	Never	1 single once	Rarely	Each month	Each week	Every day
**Cannabis**	☐	☐	☐	☐	☐	☐
**Ecstasy**	☐	☐	☐	☐	☐	☐
**Cocaine**	☐	☐	☐	☐	☐	☐
**Heroin**	☐	☐	☐	☐	☐	☐
**Speed**	☐	☐	☐	☐	☐	☐
**LSD**	☐	☐	☐	☐	☐	☐
**Other (please specify)**	☐	☐	☐	☐	☐	☐

I do not wish to answer ☐

A few questions about your home habits

Electrical and communications equipment

15. How many electrical and communication devices (telephone, alarm clock, computer, etc.) do you have in your bedroom?

☐ None ☐ 1 to 3 ☐ 4 to 6 ☐ More than 6

16. How many devices that emit radio waves, such as telephones and WiFi-connected devices, do you have in your bedroom?

☐ None ☐ 1 to 3 ☐ 4 to 6 ☐ more than 6

17. Do you have a WiFi box or relay in your bedroom?

☐ Yes ☐ No

18. Do you regularly use (>1h per week) any of the following devices (multiple answers possible) :

☐ Electric radiator (close proximity < 40 cm)

☐ Drill ☐ Hair dryer

☐ Induction stoves ☐ Magnetic mattresses

☐ Electric bed-heaters (e.g. electric blanket, waterbed) ☐ No

19. How often do you use your cell phone?

☐ I don't have a cell phone ☐ Less than an hour a day

☐ 1 to 2 hours a day ☐ 2 to 3h per day ☐ 3 to 4h per day ☐ 4 to 5h per day

☐ More than 5h per day

20. Over the past week, how much time in total have you spent on the phone without an earpiece (with the phone pressed against your ear)?

☐ I don't have a cell phone

☐ < 1 minute per day ☐ 1-10 minutes per day ☐ 10 - 20 minutes per day

☐ 20 - 40 min per day ☐ 40 min - 60 min per day ☐ 1h - 2h per day

☐ > 2h per day

21. What is your main use (which represents the most time spent on your cell phone)?

☐ Phone call ☐ Media ☐ Social network

☐ Games ☐ Utilities ☐ Others

22. On average, how long before going to sleep do you stop using screens (telephone, TV, computer, etc.)?

☐ Less than one hour ☐ 1 to 2h ☐ 2 to 3h ☐ 3 to 4h ☐ 4 to 5h ☐ More than 5h

23. Have you ever received an electric shock?

☐ Yes ☐ No

Your physical activity→ Scale: International Physical Activity Questionnaire (IPAQ)

We are interested in finding out about the kinds of physical activities that people do as part of their everyday lives. The questions will ask you about the time you spent being physically active in the **last 7 days**. Please answer each question even if you do not consider yourself to be an active person. Please think about the activities you do at work, as part of your house and yard work, to get from place to place, and in your spare time for recreation, exercise or sport.

1. Think about all the **vigorous** activities that you did in the **last 7 days**. **Vigorous** physical activities refer to activities that take hard physical effort and make you breathe much harder than normal. Think only about those physical activities that you did for at least 10 minutes at a time.

1.1 During the **last 7 days**, on how many days did you do **vigorous** physical activities like heavy lifting, digging, aerobics, or fast bicycling?

☐☐ Day(s) per week

☐ No vigorous physical activities ➔ **Skip to Q2**

1.2 How much time did you usually spend doing vigorous physical activities on one of those days?

HH : MM -☐☐ :☐☐ / day ☐ Don't know/Not sure

2. Think about all the **moderate** activities that you did in the **last 7 days**. **Moderate** activities refer to activities that take moderate physical effort and make you breathe somewhat harder than normal. Think only about those physical activities that you did for at least 10 minutes at a time.

2.1. During the **last 7 days**, on how many days did you do **moderate** physical activities like carrying light loads, bicycling at a regular pace, or doubles tennis? Do not include walking.

☐ Day(s) per week

☐ No moderate physical activities ➔ **Go to Q3**

2.2 How much time did you usually spend doing **moderate** physical activities on one of those days?

HH : MM -☐☐ :☐☐ / day ☐ Don't know/Not sure

3. Think about the time you spent **walking** in the **last 7 days**. This includes at work and at home, walking to travel from place to place, and any other walking that you have done solely for recreation, sport, exercise, or leisure.

3.1 During the **last 7 days**, on how many days did you **walk** for at least 10 minutes at a time?

☐ Day(s) per week

☐ No walking ➔ **Skip to Q4**

3.2 How much time did you usually spend **walking** on one of those days?

HH : MM -☐☐ :☐☐ / day ☐ Don't know/Not sure

4. The last question is about the time you spent **sitting** on weekdays during the **last 7 days**. Include time spent at work, at home, while doing course work and during leisure time. This may include time spent sitting at a desk, visiting friends, reading, or sitting or lying down to watch television.

4.1 During the **last 7 days**, how much time did you spend **sitting** on a **week day**?

HH : MM -☐☐ :☐☐ / day ☐ Don't know/Not sure

Your stress→ Scale: Perceived Stress Scale (PSS)

1. **The following questions ask about your <u>feelings and thoughts</u> in the past month. In each case, you will be asked to indicate how often you have felt or thought in a certain way.** Some of the questions are similar, but their value is different. Answer them as quickly as possible, and don't try to count the number of times you've felt a certain way. *Only one answer per line.*

	Never	Almost never	Sometimes	Fairly often	Very Often
**In the last month, how often have you been upset because of something that happened unexpectedly?**	☐	☐	☐	☐	☐
**In the last month, how often have you felt that you were unable to control the important things in your life?**	☐	☐	☐	☐	☐
**In the last month, how often have you felt nervous and stressed?**	☐	☐	☐	☐	☐
**In the last month, how often have you felt confident about your ability to handle your personal problems?**	☐	☐	☐	☐	☐
**In the last month, how often have you felt that things were going your way?**	☐	☐	☐	☐	☐
**In the last month, how often have you found that you could not cope with all the things that you had to do?**	☐	☐	☐	☐	☐
**In the last month, how often have you been able to control irritations in your life?**	☐	☐	☐	☐	☐
**In the last month, how often have you felt that you were on top of things?**	☐	☐	☐	☐	☐
**In the last month, how often have you been angered because of things that happened that were outside of your control?**	☐	☐	☐	☐	☐

**In the last month, how often have you felt difficulties were piling up so high that you could not overcome them?**	☐	☐	☐	☐	☐

Depression→ BDI-II scale (Beck)

97. Here you will find 21 groups of questions. For each group, choose the answer that best describes how you've been feeling <u>over the past two weeks</u>, including today. If several statements in the group seem to apply equally well, circle the highest number for that group. (*only one answer per group).*

**Sadness**	
0. I do not feel sad.	☐
1. I feel sad much of the time.	☐
2. I am sad all the time.	☐
3. I am so sad or unhappy that I can't stand it.	☐
**Pessimism**	
0. I am not discouraged about my future.	☐
1. I feel more discouraged about my future than I used to.	☐
2. I do not expect things to work out for me.	☐
3. I feel my future is hopeless and will only get worse.	☐
**Past Failures**	
0. I do not feel like a failure.	☐
1. I have failed more than I should have.	☐
2. As I look back, I see a lot of failures.	☐
3. I feel I am a total failure as a person.	☐
**Loss of Pleasure**	
0. I get as much pleasure as I ever did from the things I enjoy.	☐
1. I don't enjoy things as much as I used to.	☐
2. I get very little pleasure from the things I used to enjoy.	☐
3. I can't get any pleasure from the things I used to enjoy.	☐
**Guilty Feelings**	
0. I don't feel particularly guilty.	☐
1. I feel guilty over many things I have done or should have done.	☐
2. I feel quite guilty most of the time.	☐
3. I feel guilty all the time.	☐
**Punishment Feelings**	
0. I don't feel I am being punished.	☐
1. I feel I may be punished.	☐
2. I expect to be punished.	☐
3. I feel I am being punished.	☐
**Self Dislike**	
0. I feel the same about myself as ever.	☐
1. I have lost confidence in myself.	☐
2. I am disappointed in myself.	☐
3. I dislike myself.	☐
**Self-Criticalness**	
0. I don't criticize or blame myself more than usual.	☐
1. I am more critical of myself than I used to be.	☐
2. I criticize myself for all of my faults.	☐
3. I blame myself for everything bad that happens.	☐
**Suicidal Thoughts or Wishes**	
0. I don't have any thoughts of killing myself.	☐
1. I have thoughts of killing myself, but I would not carry them out.	☐

2. I would like to kill myself.	☐
3. I would kill myself if I had the chance.	☐
**Crying**	
0. I don't cry anymore than I used to.	☐
1. I cry more than I used to.	☐
2. I cry over every little thing.	☐
3. I feel like crying, but I can't.	☐
**Agitation**	
0. I am no more restless or wound up than usual.	☐
1. I feel more restless or wound up than usual.	☐
2. I am so restless or agitated, it's hard to stay still.	☐
3. I am so restless or agitated that I have to keep moving or doing something.	☐
**Loss of Interest**	
0. I have not lost interest in other people or activities.	☐
1. I am less interested in other people or things than before.	☐
2. I have lost most of my interest in other people or things.	☐
3. It's hard to get interested in anything.	☐
**Indecisiveness**	
0. I make decisions about as well as ever.	☐
1. I find it more difficult to make decisions than usual.	☐
2. I have much greater difficulty in making decisions than I used to.	☐
3. I have trouble making any decisions.	☐
**Worthlessness**	
0. I do not feel I am worthless.	☐
1. I don't consider myself as worthwhile and useful as I used to.	☐
2. I feel more worthless as compared to others.	☐
3. I feel utterly worthless.	☐
**Loss of Energy**	
0. I have as much energy as ever.	☐
1. I have less energy than I used to have.	☐
2. I don't have enough energy to do very much.	☐
3. I don't have enough energy to do anything.	☐
**Changes in Sleeping Pattern**	
0. I have not experienced any change in my sleeping.	☐
1a. I sleep somewhat more than usual.	☐
1b. I sleep somewhat less than usual.	☐
2a. I sleep a lot more than usual.	☐
2b. I sleep a lot less than usual.	☐
3a. I sleep most of the day.	☐
3b. I wake up 1-2 hours early and can't get back to sleep.	☐

**Irritability**	
0. I am not more irritable than usual.	☐
1. I am more irritable than usual.	☐
2. I am much more irritable than usual.	☐
3. I am irritable all the time.	☐

**Changes in Appetite**	
0. I have not experienced any change in my appetite.	☐
1a. My appetite is somewhat less than usual.	☐
1b. My appetite is somewhat greater than usual.	☐
2a. My appetite is much less than before.	☐
2b. My appetite is much greater than usual.	☐
3a. I have no appetite at all.	☐
3b. I crave food all the time.	☐
**Concentration Difficulty**	
0. I can concentrate as well as ever.	☐
1. I can't concentrate as well as usual.	☐
2. It's hard to keep my mind on anything for very long.	☐
3. I find I can't concentrate on anything.	☐
**Tiredness or Fatigue**	
0. I am no more tired or fatigued than usual.	☐
1. I get more tired or fatigued more easily than usual.	☐
2. I am too tired or fatigued to do a lot of the things I used to do.	☐
3. I am too tired or fatigued to do most of the things I used to do.	☐
**Loss of interest in sex**	
0. I have not noticed any recent change in my interest in sex.	☐
1. I am less interested in sex than I used to be.	☐
2. I am much less interested in sex now.	☐
3. I have lost interest in sex completely.	☐

Medical history→ Swiss Health Study (SHeS)

Medical history

Below is a list of diseases. Please indicate for which you have been diagnosed.

☐ ***Musculoskeletal diseases***

☐ ***Heart Diseases***

☐ ***Metabolism diseases (e.g. diabetes or cholesterol)***

☐ ***Neurological and psychological diseases***

☐ ***Respiratory health***

☐ ***Auditory health***

☐ ***Eye health***

☐ ***Kidney, liver, stomach and intestinal diseases***

☐ ***Skin diseases***

☐ ***Allergies***

☐ ***Cancers (including prostate)***

☐ ***Gynecological diseases***

☐ ***Other illnesses and rare diseases***

..

MUSCULOSKELETAL DISEASES		Have you been diagnosed by a doctor?	In what year was your illness diagnosed?	Are you currently taking treatment for this disease?	Do you see a doctor regularly for this condition?	How many times have you consulted a doctor in the last 12 months for this condition?	Have you been hospitalized for this disease?	How many nights have you spent in hospital for this illness in the last 12 months?
1	Herniated disc	☐ Yes → ☐ No	☐☐☐☐	☐ Yes ☐ No	☐ Yes ☐ No	☐☐☐	☐ Yes ☐ No	☐☐☐
2	Osteoporosis	☐ Yes → ☐ No	☐☐☐☐	☐ Yes ☐ No	☐ Yes ☐ No	☐☐☐	☐ Yes ☐ No	☐☐☐
3	Osteoarthritis	☐ Yes → ☐ No	☐☐☐☐	☐ Yes ☐ No	☐ Yes ☐ No	☐☐☐	☐ Yes ☐ No	☐☐☐

☐ I do not wish to answer

HEART DISEASES		Have you been diagnosed by a doctor?	In what year was your illness diagnosed?	Are you currently taking treatment for this disease?	Do you see a doctor regularly for this condition?	How many times have you consulted a doctor in the last 12 months for this condition?	Have you been hospitalized for this disease?	How many nights have you spent in hospital for this illness in the last 12 months?
4	Myocardial infarction (heart attack)	☐ Yes → ☐ No	☐☐☐☐	☐ Yes ☐ No	☐ Yes ☐ No	☐☐☐	☐ Yes ☐ No	☐☐☐
5	Angina pectoris, coronary artery disease	☐ Yes → ☐ No	☐☐☐☐	☐ Yes ☐ No	☐ Yes ☐ No	☐☐☐	☐ Yes ☐ No	☐☐☐
6	Heart failure	☐ Yes →	☐☐☐☐	☐ Yes	☐ Yes	☐☐☐	☐ Yes	☐☐☐

		☐ No		☐ No	☐ No		☐ No	
7	**Heart rhythm disorders (pacemaker)**	☐ Yes → ☐ No	☐☐☐☐	☐ Yes ☐ No	☐ Yes ☐ No	☐☐☐	☐ Yes ☐ No	☐☐☐
8	**Obliterative arteriopathy of the lower limbs**	☐ Yes → ☐ No	☐☐☐☐	☐ Yes ☐ No	☐ Yes ☐ No	☐☐☐	☐ Yes ☐ No	☐☐☐
9	**Hypertension**	☐ Yes → ☐ No	☐☐☐☐	☐ Yes ☐ No	☐ Yes ☐ No	☐☐☐	☐ Yes ☐ No	☐☐☐

☐ I do not wish to answer

Z 4.1 If you have already had a heart attack, how many heart attacks have you had?

☐☐ Heart attack(s)

Z 4.2. At what age did you have your first heart attack?

☐☐ Year

Z 4.3. At what age did you have your last heart attack?

☐☐ Year

	**METABOLISM DISORDERS**	**Have you been diagnosed by a doctor?**	**In what year was your illness diagnosed?**	**Are you currently taking treatment for this disease?**	**Do you see a doctor regularly for this condition?**	**How many times have you consulted a doctor in the last 12 months for this condition?**	**Have you been hospitalized for this disease?**	**How many nights have you spent in hospital for this illness in the last 12 months?**
**10a**	**Type I diabetes mellitus**	☐ Yes → ☐ No	☐☐☐☐	☐ Yes ☐ No	☐ Yes ☐ No	☐☐☐	☐ Yes ☐ No	☐☐☐
**10b**	**Type II diabetes mellitus**	☐ Yes →	☐☐☐☐	☐ Yes	☐ Yes	☐☐☐	☐ Yes	☐☐☐

		☐ No		☐ No	☐ No		☐ No	
10c	Gestational diabetes (during pregnancy)	☐ Yes → ☐ No	☐☐☐☐	☐ Yes ☐ No	☐ Yes ☐ No	☐☐☐	☐ Yes ☐ No	☐☐☐
11	Increased blood lipids, cholesterol or triglycerides	☐ Yes → ☐ No	☐☐☐☐	☐ Yes ☐ No	☐ Yes ☐ No	☐☐☐	☐ Yes ☐ No	☐☐☐
12	Drop	☐ Yes → ☐ No	☐☐☐☐	☐ Yes ☐ No	☐ Yes ☐ No	☐☐☐	☐ Yes ☐ No	☐☐☐
13a	Hyperthyroidism	☐ Yes → ☐ No	☐☐☐☐	☐ Yes ☐ No	☐ Yes ☐ No	☐☐☐	☐ Yes ☐ No	☐☐☐
13b	Hypothyroidism	☐ Yes → ☐ No	☐☐☐☐	☐ Yes ☐ No	☐ Yes ☐ No	☐☐☐	☐ Yes ☐ No	☐☐☐
13c	Other thyroid disorders : ___________	☐ Yes → ☐ No	☐☐☐☐	☐ Yes ☐ No	☐ Yes ☐ No	☐☐☐	☐ Yes ☐ No	☐☐☐

☐ I do not wish to answer

If you have diabetes, please answer questions Z 10.1 to Z 10.3.

Z 10.1. How are you currently treated (including insulin injections and insulin pumps)?

- ☐ Insulin only
- ☐ Tablets only
- ☐ Insulin and tablets
- ☐ Dietary treatment only
- ☐ Other treatment
- ☐ I do not take any treatment
- ☐ Don't know

Z 10.2. If you are taking insulin, from what age have you been taking it?

☐☐ years

Z 10.2. How are you currently treated (including insulin injections and insulin pumps)?

☐ Retinopathy, corneal damage
☐ Blindness
☐ Renal failure or insufficiency
☐ Dialysis treatment or kidney transplantation
☐ Diabetic foot (poor wound healing)
☐ Amputations (toe, foot, leg)
☐ None of these diseases or complications
☐ Don't know

	NEUROLOGICAL DISEASES AND PSYCHICS	Have you been diagnosed by a doctor?	In what year was your illness diagnosed?	Are you currently taking treatment for this disease?	Do you see a doctor regularly for this condition?	How many times have you consulted a doctor in the last 12 months for this condition?	Have you been hospitalized for this disease?	How many nights have you spent in hospital for this illness in the last 12 months?
14	Stroke	☐ Yes → ☐ No	☐☐☐☐	☐ Yes ☐ No	☐ Yes ☐ No	☐☐☐	☐ Yes ☐ No	☐☐☐
15	Epilepsy	☐ Yes → ☐ No	☐☐☐☐	☐ Yes ☐ No	☐ Yes ☐ No	☐☐☐	☐ Yes ☐ No	☐☐☐
16	Migraines	☐ Yes → ☐ No	☐☐☐☐	☐ Yes ☐ No	☐ Yes ☐ No	☐☐☐	☐ Yes ☐ No	☐☐☐
17	Parkinson's syndrome	☐ Yes → ☐ No	☐☐☐☐	☐ Yes ☐ No	☐ Yes ☐ No	☐☐☐	☐ Yes ☐ No	☐☐☐
18	Depression	☐ Yes → ☐ No	☐☐☐☐	☐ Yes ☐ No	☐ Yes ☐ No	☐☐☐	☐ Yes ☐ No	☐☐☐
19	Anxiety disorders or panic attacks	☐ Yes →	☐☐☐☐	☐ Yes	☐ Yes	☐☐☐	☐ Yes	☐☐☐

		☐ No		☐ No	☐ No		☐ No	
20	Eating disorders	☐ Yes → ☐ No	☐☐☐☐	☐ Yes ☐ No	☐ Yes ☐ No	☐☐☐	☐ Yes ☐ No	☐☐☐
21	Attention deficit disorder with or without hyperactivity (ADHD)	☐ Yes → ☐ No	☐☐☐☐	☐ Yes ☐ No	☐ Yes ☐ No	☐☐☐	☐ Yes ☐ No	☐☐☐
22	Dementia or Alzheimer's	☐ Yes → ☐ No	☐☐☐☐	☐ Yes ☐ No	☐ Yes ☐ No	☐☐☐	☐ Yes ☐ No	☐☐☐
23	Amyotrophic lateral sclerosis (ALS)	☐ Yes → ☐ No	☐☐☐☐	☐ Yes ☐ No	☐ Yes ☐ No	☐☐☐	☐ Yes ☐ No	☐☐☐
24	Multiple sclerosis	☐ Yes → ☐ No	☐☐☐☐	☐ Yes ☐ No	☐ Yes ☐ No	☐☐☐	☐ Yes ☐ No	☐☐☐

☐ I do not wish to answer

If you have or have had an eating disorder, please answer the following question (Z 20.1)

Z 20.1 What type of eating disorder is/was involved?

Multiple answers possible

☐ Anorexia ☐ Bulimia ☐ Binge-Eating ☐ Other ______________

	PULMONARY ILLNESSES	Have you been diagnosed by a doctor?	In what year was your illness diagnosed?	Are you currently taking treatment for this disease?	Do you see a doctor regularly for this condition?	How many times have you consulted a doctor in the last 12 months for this condition?	Have you been hospitalized for this disease?	How many nights have you spent in hospital for this illness in the last 12 months?
25	Asthma	☐ Yes → ☐ No	☐☐☐☐	☐ Yes ☐ No	☐ Yes ☐ No	☐☐☐	☐ Yes ☐ No	☐☐☐
26	Chronic bronchitis	☐ Yes → ☐ No	☐☐☐☐	☐ Yes ☐ No	☐ Yes ☐ No	☐☐☐	☐ Yes ☐ No	☐☐☐
27	Chronic obstructive pulmonary disease (COPD)	☐ Yes → ☐ No	☐☐☐☐	☐ Yes ☐ No	☐ Yes ☐ No	☐☐☐	☐ Yes ☐ No	☐☐☐
28	Pulmonary emphysema	☐ Yes → ☐ No	☐☐☐☐	☐ Yes ☐ No	☐ Yes ☐ No	☐☐☐	☐ Yes ☐ No	☐☐☐
29	Sleep apnea	☐ Yes → ☐ No	☐☐☐☐	☐ Yes ☐ No	☐ Yes ☐ No	☐☐☐	☐ Yes ☐ No	☐☐☐
30	Pneumonia	☐ Yes → ☐ No	☐☐☐☐	☐ Yes ☐ No	☐ Yes ☐ No	☐☐☐	☐ Yes ☐ No	☐☐☐
31	Other lung diseases:	☐ Yes → ☐ No	☐☐☐☐	☐ Yes ☐ No	☐ Yes ☐ No	☐☐☐	☐ Yes ☐ No	☐☐☐

☐ I do not wish to answer

If you have ever had asthma (even if not diagnosed by a doctor), please answer questions Z 25.1 to Z 25.3.

Z 25.1 Have you had one or more asthma attacks in the last 12 months?

☐ Yes ☐ No ☐ I don't know

Z 25.2 If yes, how many attacks have you had in the last 12 months?

Enter even an approximate number

☐☐☐ Asthma attack(s)

Z 25.3 Are you currently taking asthma medication (also inhalers, aerosols, tablets)?

☐ Yes ☐ No ☐ I don't know

HEARING DISORDERS	Have you been diagnosed by a doctor?	In what year was your illness diagnosed?	Are you currently taking treatment for this disease?	Do you see a doctor regularly for this condition?	How many times have you consulted a doctor in the last 12 months for this condition?	Have you been hospitalized for this disease?	How many nights have you spent in hospital for this illness in the last 12 months?
**32 Tinnitus**	☐ Yes → ☐ No	☐☐☐☐	☐ Yes ☐ No	☐ Yes ☐ No	☐☐☐	☐ Yes ☐ No	☐☐☐
**33 Hearing loss**	☐ Yes → ☐ No	☐☐☐☐	☐ Yes ☐ No	☐ Yes ☐ No	☐☐☐	☐ Yes ☐ No	☐☐☐
**34 Hearing disorders**	☐ Yes → ☐ No	☐☐☐☐	☐ Yes ☐ No	☐ Yes ☐ No	☐☐☐	☐ Yes ☐ No	☐☐☐
**35 Balance disorders / Vertigo**	☐ Yes → ☐ No	☐☐☐☐	☐ Yes ☐ No	☐ Yes ☐ No	☐☐☐	☐ Yes ☐ No	☐☐☐

☐ I do not wish to answer

36. Do you wear a hearing aid?

☐ Yes ☐ No

36.1 If yes to 36 which ear(s)?

☐ Right ☐ Left ☐ Both ears

36.2 If yes, how long have you been wearing your hearing aid(s)?

☐☐ year

EYE DISEASES	Have you been diagnosed by a doctor?	In what year was your illness diagnosed?	Are you currently taking treatment for this disease?	Do you see a doctor regularly for this condition?	How many times have you consulted a doctor in the last 12 months for this condition?	Have you been hospitalized for this disease?	How many nights have you spent in hospital for this illness in the last 12 months?
**37 Cataracts**	☐ Yes → ☐ No	☐☐☐☐	☐ Yes ☐ No	☐ Yes ☐ No	☐☐☐	☐ Yes ☐ No	☐☐☐
**38 Glaucoma**	☐ Yes → ☐ No	☐☐☐☐	☐ Yes ☐ No	☐ Yes ☐ No	☐☐☐	☐ Yes ☐ No	☐☐☐
**39 Age-related macular degeneration (AMD)**	☐ Yes → ☐ No	☐☐☐☐	☐ Yes ☐ No	☐ Yes ☐ No	☐☐☐	☐ Yes ☐ No	☐☐☐
**40 Other eye disease:____________**	☐ Yes → ☐ No	☐☐☐☐	☐ Yes ☐ No	☐ Yes ☐ No	☐☐☐	☐ Yes ☐ No	☐☐☐

☐ I do not wish to answer

41. Do you wear glasses or contact lenses?

☐ Yes ☐ No

41.1 If yes to 80.1 or yes to 80.2, what is your current correction? :

Right eye : Myopia (-) ☐☐☐

Hyperopia (+) ☐☐☐

Astigmatism ☐ Yes ☐ No

Left eye : Myopia (-) ☐☐☐

Hyperopia (+) ☐☐☐

Astigmatism ☐ Yes ☐ No

Presbyopia ☐ Yes ☐ No

41.2 How long have you worn glasses or contact lenses?

☐☐ Years

KIDNEY, LIVER, STOMACH, INTESTINS DISEASES	Have you been diagnosed by a doctor?	In what year was your illness diagnosed?	Are you currently taking treatment for this disease?	Do you see a doctor regularly for this condition?	How many times have you consulted a doctor in the last 12 months for this condition?	Have you been hospitalized for this disease?	How many nights have you spent in hospital for this illness in the last 12 months?
**42 Gastric or duodenal ulcer**	☐ Yes → ☐ No	☐☐☐☐	☐ Yes ☐ No	☐ Yes ☐ No	☐☐☐	☐ Yes ☐ No	☐☐☐
**43 Heartburn or upset stomach**	☐ Yes → ☐ No	☐☐☐☐	☐ Yes ☐ No	☐ Yes ☐ No	☐☐☐	☐ Yes ☐ No	☐☐☐

**44 Intestinal inflammation (e.g. ulcerative colitis or Crohn's disease)**	☐ Yes → ☐ No	☐☐☐☐	☐ Yes ☐ No	☐ Yes ☐ No	☐☐☐	☐ Yes ☐ No	☐☐☐
**45 Gallstones**	☐ Yes → ☐ No	☐☐☐☐	☐ Yes ☐ No	☐ Yes ☐ No	☐☐☐	☐ Yes ☐ No	☐☐☐
**46 Cirrhosis of the liver**	☐ Yes → ☐ No	☐☐☐☐	☐ Yes ☐ No	☐ Yes ☐ No	☐☐☐	☐ Yes ☐ No	☐☐☐
**47 Kidney stones, in the ureter or bladder**	☐ Yes → ☐ No	☐☐☐☐	☐ Yes ☐ No	☐ Yes ☐ No	☐☐☐	☐ Yes ☐ No	☐☐☐
**48 Impaired renal function (e.g. chronic renal failure)**	☐ Yes → ☐ No	☐☐☐☐	☐ Yes ☐ No	☐ Yes ☐ No	☐☐☐	☐ Yes ☐ No	☐☐☐

☐ I do not wish to answer

If you have ever had kidney failure, please answer the following question.

Z 48.1. Have you ever had dialysis?

☐ Yes ☐ No ☐ I don't know

**SKIN DISORDERS**	**Have you been diagnosed by a doctor?**	**In what year was your illness diagnosed?**	**Are you currently taking treatment for this disease?**	**Do you see a doctor regularly for this condition?**	**How many times have you consulted a doctor in the last 12 months for this condition?**	**Have you been hospitalized for this disease?**	**How many nights have you spent in hospital for this illness in the last 12 months?**
**49 Atopic dermatitis (neurodermatitis) or eczema**	☐ Yes → ☐ No	☐☐☐☐	☐ Yes ☐ No	☐ Yes ☐ No	☐☐☐	☐ Yes ☐ No	☐☐☐
**50 Psoriasis**	☐ Yes → ☐ No	☐☐☐☐	☐ Yes ☐ No	☐ Yes ☐ No	☐☐☐	☐ Yes ☐ No	☐☐☐

**51 Severe acne**	☐ Yes → ☐ No	☐☐☐☐	☐ Yes ☐ No	☐ Yes ☐ No	☐☐☐	☐ Yes ☐ No	☐☐☐

☐ I do not wish to answer

**ALLERGIES**	**Have you been diagnosed by a doctor?**	**In what year was your illness diagnosed?**	**Are you currently taking treatment for this disease?**	**Do you see a doctor regularly for this condition?**	**How many times have you consulted a doctor in the last 12 months for this condition?**	**Have you been hospitalized for this disease?**	**How many nights have you spent in hospital for this illness in the last 12 months?**
**52 Hay fever**	☐ Yes → ☐ No	☐☐☐☐	☐ Yes ☐ No	☐ Yes ☐ No	☐☐☐	☐ Yes ☐ No	☐☐☐
**53 Other allergic colds**	☐ Yes → ☐ No	☐☐☐☐	☐ Yes ☐ No	☐ Yes ☐ No	☐☐☐	☐ Yes ☐ No	☐☐☐
**54 Allergy to insect stings (bee, wasp, bumblebee, hornet)**	☐ Yes → ☐ No	☐☐☐☐	☐ Yes ☐ No	☐ Yes ☐ No	☐☐☐	☐ Yes ☐ No	☐☐☐
**55 Dust mite allergy**	☐ Yes → ☐ No	☐☐☐☐	☐ Yes ☐ No	☐ Yes ☐ No	☐☐☐	☐ Yes ☐ No	☐☐☐
**56 Animal hair allergy**	☐ Yes → ☐ No	☐☐☐☐	☐ Yes ☐ No	☐ Yes ☐ No	☐☐☐	☐ Yes ☐ No	☐☐☐
**57 Contact allergies (latex, nickel, perfumes, etc.)**	☐ Yes → ☐ No	☐☐☐☐	☐ Yes ☐ No	☐ Yes ☐ No	☐☐☐	☐ Yes ☐ No	☐☐☐
**58 Chronic sinusitis**	☐ Yes → ☐ No	☐☐☐☐	☐ Yes ☐ No	☐ Yes ☐ No	☐☐☐	☐ Yes ☐ No	☐☐☐
**59 Food allergy (specify) : ____**	☐ Yes → ☐ No	☐☐☐☐	☐ Yes ☐ No	☐ Yes ☐ No	☐☐☐	☐ Yes ☐ No	☐☐☐
**60 Food intolerance (specify) :___**	☐ Yes →	☐☐☐☐	☐ Yes	☐ Yes	☐☐☐	☐ Yes	☐☐☐

	☐ No		☐ No	☐ No		☐ No	
61 Other allergy (specify):___	☐ Yes → ☐ No	☐☐☐☐	☐ Yes ☐ No	☐ Yes ☐ No	☐☐☐	☐ Yes ☐ No	☐☐☐

CANCER	Have you been diagnosed by a doctor?	In what year was your illness diagnosed?	Are you currently taking treatment for this disease?	Do you see a doctor regularly for this condition?	How many times have you consulted a doctor in the last 12 months for this condition?	Have you been hospitalized for this disease?	How many nights have you spent in hospital for this illness in the last 12 months?
**62 Lung**	☐ Yes → ☐ No	☐☐☐☐	☐ Yes ☐ No	☐ Yes ☐ No	☐☐☐	☐ Yes ☐ No	☐☐☐
**63 Colorectal**	☐ Yes → ☐ No	☐☐☐☐	☐ Yes ☐ No	☐ Yes ☐ No	☐☐☐	☐ Yes ☐ No	☐☐☐
**64 Stomach**	☐ Yes → ☐ No	☐☐☐☐	☐ Yes ☐ No	☐ Yes ☐ No	☐☐☐	☐ Yes ☐ No	☐☐☐
**65 Esophagus**	☐ Yes → ☐ No	☐☐☐☐	☐ Yes ☐ No	☐ Yes ☐ No	☐☐☐	☐ Yes ☐ No	☐☐☐
**66 Hepatocellular carcinoma**	☐ Yes → ☐ No	☐☐☐☐	☐ Yes ☐ No	☐ Yes ☐ No	☐☐☐	☐ Yes ☐ No	☐☐☐
**67 Sein**	☐ Yes → ☐ No	☐☐☐☐	☐ Yes ☐ No	☐ Yes ☐ No	☐☐☐	☐ Yes ☐ No	☐☐☐
**68 Glioma**	☐ Yes → ☐ No	☐☐☐☐	☐ Yes ☐ No	☐ Yes ☐ No	☐☐☐	☐ Yes ☐ No	☐☐☐

**69 Meningioma**	☐ Yes → ☐ No	☐☐☐☐	☐ Yes ☐ No	☐ Yes ☐ No	☐☐☐	☐ Yes ☐ No	☐☐☐
**70 Women: cervix**	☐ Yes → ☐ No	☐☐☐☐	☐ Yes ☐ No	☐ Yes ☐ No	☐☐☐	☐ Yes ☐ No	☐☐☐
**71 Men: prostate**	☐ Yes → ☐ No	☐☐☐☐	☐ Yes ☐ No	☐ Yes ☐ No	☐☐☐	☐ Yes ☐ No	☐☐☐
**72 Leukemia**	☐ Yes → ☐ No	☐☐☐☐	☐ Yes ☐ No	☐ Yes ☐ No	☐☐☐	☐ Yes ☐ No	☐☐☐
**73 Hodgkin's disease**	☐ Yes → ☐ No	☐☐☐☐	☐ Yes ☐ No	☐ Yes ☐ No	☐☐☐	☐ Yes ☐ No	☐☐☐
**74 Cutaneous melanomas**	☐ Yes → ☐ No	☐☐☐☐	☐ Yes ☐ No	☐ Yes ☐ No	☐☐☐	☐ Yes ☐ No	☐☐☐
**75 Salivary gland tumors**	☐ Yes → ☐ No	☐☐☐☐	☐ Yes ☐ No	☐ Yes ☐ No	☐☐☐	☐ Yes ☐ No	☐☐☐
**76 Pituitary adenoma**	☐ Yes → ☐ No	☐☐☐☐	☐ Yes ☐ No	☐ Yes ☐ No	☐☐☐	☐ Yes ☐ No	☐☐☐
**77 Malignant tumor of the eye and adnexa**	☐ Yes → ☐ No	☐☐☐☐	☐ Yes ☐ No	☐ Yes ☐ No	☐☐☐	☐ Yes ☐ No	☐☐☐
**78 Other cancer (specify) : __________**	☐ Yes → ☐ No	☐☐☐☐	☐ Yes ☐ No	☐ Yes ☐ No	☐☐☐	☐ Yes ☐ No	☐☐☐
**79 Other cancer (specify) : ___________**	☐ Yes → ☐ No	☐☐☐☐	☐ Yes ☐ No	☐ Yes ☐ No	☐☐☐	☐ Yes ☐ No	☐☐☐

☐ I do not wish to answer

	GYNAECOLOGICAL DISEASES	Have you been diagnosed by a doctor?	In what year was your illness diagnosed?	Are you currently taking treatment for this disease?	Do you see a doctor regularly for this condition?	How many times have you consulted a doctor in the last 12 months for this condition?	Have you been hospitalized for this disease?	How many nights have you spent in hospital for this illness in the last 12 months?
80	Polycystic ovary syndrome (Stein-Leventhal syndrome)	☐ Yes → ☐ No	☐☐☐☐	☐ Yes ☐ No	☐ Yes ☐ No	☐☐☐	☐ Yes ☐ No	☐☐☐
81	Endometriosis	☐ Yes → ☐ No	☐☐☐☐	☐ Yes ☐ No	☐ Yes ☐ No	☐☐☐	☐ Yes ☐ No	☐☐☐
82	Uterine myoma (benign tumor)	☐ Yes → ☐ No	☐☐☐☐	☐ Yes ☐ No	☐ Yes ☐ No	☐☐☐	☐ Yes ☐ No	☐☐☐
83	Uterine prolapse (or uterine descent)	☐ Yes → ☐ No	☐☐☐☐	☐ Yes ☐ No	☐ Yes ☐ No	☐☐☐	☐ Yes ☐ No	☐☐☐
84	Papillomavirus or HPV infection (in the cervix)	☐ Yes → ☐ No	☐☐☐☐	☐ Yes ☐ No	☐ Yes ☐ No	☐☐☐	☐ Yes ☐ No	☐☐☐

☐ I do not wish to answer

	OTHER ILLNESSES AND RARE DISEASES	Have you been diagnosed by a doctor?	In what year was your illness diagnosed?	Are you currently taking treatment for this disease?	Do you see a doctor regularly for this condition?	How many times have you consulted a doctor in the last 12 months for this condition?	Have you been hospitalized for this disease?	How many nights have you spent in hospital for this illness in the last 12 months?
85	**Urinary tract infection (UTI)**	☐ Yes → ☐ No	☐☐☐☐	☐ Yes ☐ No	☐ Yes ☐ No	☐☐☐	☐ Yes ☐ No	☐☐☐
86	**Rheumatoid arthritis / Polyarthritis**	☐ Yes → ☐ No	☐☐☐☐	☐ Yes ☐ No	☐ Yes ☐ No	☐☐☐	☐ Yes ☐ No	☐☐☐
87	**Fibromyalgia**	☐ Yes → ☐ No	☐☐☐☐	☐ Yes ☐ No	☐ Yes ☐ No	☐☐☐	☐ Yes ☐ No	☐☐☐
88	**Congenital malformation**	☐ Yes → ☐ No	☐☐☐☐	☐ Yes ☐ No	☐ Yes ☐ No	☐☐☐	☐ Yes ☐ No	☐☐☐
89	**Electrohypersensitivity**	☐ Yes → ☐ No	☐☐☐☐	☐ Yes ☐ No	☐ Yes ☐ No	☐☐☐	☐ Yes ☐ No	☐☐☐
90	**Other disease 1 :_______________**	☐ Yes → ☐ No	☐☐☐☐	☐ Yes ☐ No	☐ Yes ☐ No	☐☐☐	☐ Yes ☐ No	☐☐☐
91	**Other illness 2 :_______________**	☐ Yes → ☐ No	☐☐☐☐	☐ Yes ☐ No	☐ Yes ☐ No	☐☐☐	☐ Yes ☐ No	☐☐☐

☐ I do not wish to answer

If you have a congenital malformation, please answer the following question (Z 88.1):

Z 88.1. What type of congenital malformation is it?

Multiple answers possible

- ☐ Musculoskeletal malformations (e.g. clubfoot, hip dysplasia)
- ☐ Malformations of the internal genitourinary system (e.g. renal malformation)
- ☐ Central nervous system defects (e.g. non-closure of the neural tube)
- ☐ Malformations of the external genitourinary system (e.g. hypospadia)
- ☐ Facial clefts (e.g. cleft lip and/or palate)
- ☐ Cardiovascular deformities
- ☐ Malformations of the digestive system
- ☐ Chromosomal aberrations
- ☐ Ear deformities
- ☐ Eye deformities
- ☐ Minor morphogenesis malformations (e.g., single palmar crease, non-closure of rectus muscles, excess or fusion of fingers or toes, hemangiomas, nevi, ...)
- ☐ Other

Your Surgeries → Swiss Health Study (SHeS)

92. Have you had any of the following operations? ***Please check when the operation took place.***
Multiple answers possible.

☐ I've never had a surgery before.

☐ *Heart valve surgery*

☐ *Coronary angioplasty (PTCA) with or without stent placement*

☐ *Coronary artery bypass grafting*

☐ *Fitting a pacemaker*

☐ *Balloon dilatation of a leg vein with or without stent placement*

☐ *Peripheral leg artery bypass surgery*

☐ *Carotid artery surgery*

☐ *Refractive surgery/laser eye surgery*

☐ *Cataract surgery*

☐ *Spinal surgery (e.g. herniated disc, sciatica)*

☐ *Knee surgery*

☐ *Knee prosthesis*

☐ *Hand or arm surgery (e.g. carpal tunnel operation)*

☐ *Shoulder surgery*

☐ *Ankle or foot surgery (Hallux valgus)*

☐ *Hip surgery*

☐ *Hip prosthesis*

☐ *Appendectomy*

☐ *Spleen surgery*

☐ *Throat or tonsil surgery*

☐ *Thymus*

☐ *Surgery for polyps in the paranasal sinuses*

☐ *Gallbladder surgery*

☐ *Thyroid surgery*

☐ *Uterine surgery (woman)*

☐ *Ovarian surgery (woman)*

☐ *Left breast operation*

☐ *Right breast operation*

☐ *Prostate surgery (men)*

☐ *If Other, which one?* ____

☐ I do not wish to answer

Your treatments→ Swiss Health Study (SHeS)

93. At present, how many medications do you use regularly (i.e. at least once a week for at least one month), including painkillers, tranquilizers, sleeping pills and natural treatments (tablets, syrups, nasal sprays, eye drops, suppositories, injections, ointments, etc.)?

Please indicate the number (even approximate) of medications; if you do NOT take medication regularly, please enter 0.

☐☐☐ medications taken regularly

94. Please indicate the name and frequency of all medications you are taking (including painkillers, tranquilizers, sleeping pills and natural treatments).

Drug name (Dosage) as indicated on the box	Several times a day	1 time a day	Several times a week	1 time per week
	☐	☐	☐	☐
	☐	☐	☐	☐
	☐	☐	☐	☐
	☐	☐	☐	☐
	☐	☐	☐	☐
	☐	☐	☐	☐
	☐	☐	☐	☐
	☐	☐	☐	☐

	☐	☐	☐	☐
	☐	☐	☐	☐
	☐	☐	☐	☐
	☐	☐	☐	☐
	☐	☐	☐	☐

☐ I do not wish to answer

Family medical history

Some of the questions we're about to ask you are sensitive. They may be more difficult to answer. If you feel uncomfortable answering these questions, don't hesitate to contact an association offering support by calling 143, or by writing to www.143.ch. **This contact is completely anonymous.**

95. In the last 12 months, have you lost a biological parent?

☐ Yes, my father ☐ Yes, my mother ☐ No ☐ I don't know ☐ I don't wish to answer

If yes for my father, at what age? ☐☐☐

If yes, what was the cause of death? ____________________

If yes for my mother, at what age? ☐☐☐

If yes, what was the cause of death? ____________________

96. Have your parents been diagnosed with any of these diseases? If yes, tick the appropriate boxes and, if necessary, fill in the approximate age at diagnosis:

	**Biological father**	**Biological mother**
**Cancer**	☐	☐
Specify location	________	________
Age at diagnosis	☐☐ years	☐☐ years
Specify location	________	________
Age at diagnosis	☐☐ years	☐☐ years
**Infarction**	☐	☐
Age at diagnosis	☐☐ years	☐☐ years
**Angina pectoris**	☐	☐
Age at diagnosis	☐☐ years	☐☐ years
**Hypertension**	☐	☐
Age at diagnosis	☐☐ years	☐☐ years
**Sudden death**	☐	☐
Age	☐☐ years	☐☐ years
**AVC**	☐	☐
Age at diagnosis	☐☐ years	☐☐ years
**Alzheimer's disease**	☐	☐
Age at diagnosis	☐☐ years	☐☐ years
**Severe psychiatric illness**	☐	☐
Specify	________	________
Age at diagnosis	☐☐ years	☐☐ years
**Suicide**	☐	☐
Age	☐☐ years	☐☐ years
**Type II diabetes (NIDDM)**	☐	☐
Age at diagnosis	☐☐ years	☐☐ years
**Dialysis or kidney transplant**	☐	☐
Age	☐☐ years	☐☐ years
**Asthma**	☐	☐
Age at diagnosis	☐☐ years	☐☐ years

☐ I do not wish to answer

Women's health

(TO BE COMPLETED BY WOMEN ONLY)

Here we ask questions about gynecological health to assess the impact of working conditions on women's health, particularly in relation to stress, fatigue and irregular working hours. Your answers are **de-identified to strictly protect your anonymity**. They will never be used individually, but together with the answers of all participants. They will **never be shared with your employer.**

Some questions are sensitive and may be difficult to answer. You have the choice not to answer. If you feel uncomfortable answering these questions, don't hesitate to contact an association offering support by calling 143, or by writing to www.143.ch. **This contact is completely anonymous.**

1. At what age did you have your first period?

☐☐ years

☐ I do not wish to answer

2. When was your last period?

☐ Less than 3 months

☐ Between 3 and 12 months
☐ Over 12 months
Year ☐☐☐☐

☐ I don't know
☐ I do not wish to answer

3. According to your last 3 cycles, your periods are :
☐ Regular, naturally
☐ Regular hormone therapy (e.g. the pill)
☐ Irregular
☐ I don't know
☐ I do not wish to answer

4. On average, how long are your menstrual cycles?

☐☐ days

☐ My cycles are too irregular

☐ I do not wish to answer

5. What is the reason for the absence of your period for 3 months or more?

☐ I am in menopause ☐ I'm breastfeeding

☐ I am pregnant ☐ I'm an elite sportswoman

☐ I had my uterus removed ☐ I had 2 ovaries removed

☐ I take a continuous hormonal treatment (pill, IUD or other).

Specify : ________________________________

☐ Other situation

Specify : ________________________________

☐ I don't know

☐ I do not wish to answer

6. Are you taking or have you ever taken hormone replacement therapy such as estrogen, progestin or a combination of the 2 (e.g. to treat menopausal symptoms or absence of menstrual periods)?

Please consider all forms of treatment: tablets, creams or gels, patches, injections, suppositories or drops.

☐ No

☐ Yes, in the past

☐ Yes

☐ I don't know

☐ I do not wish to answer

7. Are you currently using hormonal contraception?
Please consider all forms of treatment: tablets, creams or gels, patches, injections, suppositories or drops.

☐ No

☐ Yes

Which one?

☐ Pill
☐ Hormonal implant
☐ Hormonal IUD
☐ Copper coil
☐ Vaginal ring
☐ Other hormone treatments

Product name : ____________________

Age: ☐☐

☐ I do not wish to answer

8. Have you ever been pregnant while actively working as a bus driver?

☐ Yes ☐ No

☐ I do not wish to answer

9. If yes, did you benefit from job accommodation or other measures during your pregnancy?

☐ Yes. Can you describe them? : ____________________

☐ No

☐ I do not wish to answer

9.1 Has your employer made any arrangements to facilitate your return to work after your pregnancy?

☐ Yes

Can you describe them? : ____________________

☐ No

☐ I do not wish to answer

Reproductive health

Some of the questions we're about to ask you are sensitive. They may be more difficult to answer. If you feel uncomfortable answering these questions, don't hesitate to contact an association offering support by calling 143, or by writing to www.143.ch. This contact is completely anonymous.

10. Do you have one or more biological children?

☐ No

☐ Yes. Total number of biological children:______________?

11. For each of your biological children, please indicate in the table below: their gender, year of birth, birth weight, whether they were breastfed (even partially) and, if so, for how many months:

Child	Gender		Year of birth	Birth weight	Born premature			Breastfeeding			Malformation (p. e.g. heart, hearing impairment, spina bifida...)
	♂	♀			Yes	No	How many weeks of amenorrhea (SA)	Yes	No	How many months (*)	
1	☐	☐	☐☐☐☐	☐☐☐☐ g	☐	☐	☐☐ weeks	☐	☐	☐☐ month	
2	☐	☐	☐☐☐☐	☐☐☐☐ g	☐	☐		☐	☐		
3	☐	☐	☐☐☐☐	☐☐☐☐ g	☐	☐		☐	☐		
4	☐	☐	☐☐☐☐	☐☐☐☐ g	☐	☐		☐	☐		
5	☐	☐	☐☐☐☐	☐☐☐☐ g	☐	☐		☐	☐		

* If you breastfed for less than a month, enter "0"; if you breastfed for between 1 and 2 months, enter "1", etc.

12. Have you experienced a miscarriage or loss of child at birth in your relationship?

☐ No

☐ Yes,

If so, how many times?

- Miscarriage ____________________________
- Child born dead / loss of child at birth ____________________________

At what week(s) of pregnancy (SA)

- Miscarriage ____________________________
- Child born dead/ / loss of child at birth ____________________________

☐ I do not wish to answer

13. Have you ever tried to conceive a child for at least 1 year (6 months if you were over 35), without success?

☐ No

☐ Yes

If so, has a medical diagnosis been made?

☐ No

☐ Yes, diagnosis : ☐ Male infertility (specify)______________

☐ Female infertility (specify) ______________

☐ Other (specify) __________________

☐ Reason unknown

☐ I do not wish to answer

YOUR PROFESSIONAL CAREER

We'll start by asking you a few questions about your professional career. The questions will cover the company you currently work for or have worked for in the past, as well as the bus models you use or have used. This information will enable us to assess your exposure to physicochemical and ergonomic risks. Please answer each question as accurately as possible.

Your career→ Bus

A FEW QUESTIONS ABOUT YOUR PROFESSIONAL CAREER

1st vehicle

1. Which company do you currently work for, or did you work for last? *(one answer only)*

3, Aargau Verkehr (AVA) | 4, Busbetrieb Aarau AG (BBA) | 5, Aare Seeland mobil | 6, Autilinea Mendriense (AMSA) | 7, Autolinee Bleniesi SA (ABL) | 8, Auto AG Group (AAGR) | 9, Auto AG Schwyz | 10, Auto Ag Uri | 11, Autobetrieb Sernftal | 12, Autobetrieb Weesen-Amden (AWA) | 13, Autobus AG Liestal (AAGL) | 14, Autokurse Oberthurgau (AOT) | 15, Autolinee Regionali Luganesi (arl) | 16, Automobilverkehr Frutigen-Adelboden (AFA) | 17, Baselland Transport (BLT) | 18, Basler Verkehrsbetriebe (BVB) | 19, Bernmobil | 20, Busbetrieb Seetal Freiamt (BSF) | 21, BUS Ostschweiz AG (BO) | 22, Bus sédunois | 23, Busbetrieb Grenchen und Umgebung (BGU) | 24, Bus Sarganserland-Werdenberg (BSW) | 25, Busbetrieb Linchtensteig-Wattwil-Ebnat-Kappel (blwe) | 26, Busbetrieb Olten Gösgen Gäu (BOGG) | 27, Busbetrieb Solothurn und Umgebung (BSU) | 28, Busland AG Langnau Burgdorf (BLS) | 29, Chemin de fer du jura | 30, Compagnie de Chemin de fer et d'autobus Sierre-Montana-Crans SA (SMC) | 31, Engadin Bus | 32, Ferrovie Autolinee Regionali Ticinesi (FART) Locarno | 33, GEM'Bus | 34, Genève tour (G-tour) | 35, Globe Limo | 36, Odier Excursion | 37, Pfosi Arosa Bus AG | 38, PostAuto Schweiz AG (Carpostal) | 39, Ratpdev | 40, RDTA | 41, Regiobus AG Gossau (Regio) | 42, Regionalbus Lenzburg(RBL) | 43, Regionale Verkehrsbetriebe Baden-Wettingen (RVBW) | 44, Regionale Verkehrsbetriebe Schaffhausen RVSH | 45, Rottal Auto AG | 46, SBB-Bus Zofingen/Reiden | 47, Schneider Busbetriebe | 48, Seebus | 49, Sihltal Zürich Uetliberg Bahn AG (SZU) | 50, Stadtbus Chur | 51, Stadtbus Frauenfeld | 52, Stadtbus Kreuzlingen | 53, Stadtbus Winterthur (SW) | 54, STI Bus AG | 55, Tellbus Altdorf-Luzern | 56, Transports de la région Morges-Bière-Cossonay (MBC) | 57, Transports Martigny Région (TMR) | 58, Transports publics de la région lausannoise (tl) | 59, Transports publics de la région nyonnaise (tpn) | 60, Transports publics du Chablais (TPC) | 61, Transports publics fribourgeois (TPF) | 62, Transports publics genevois (TPG) | 63, Transports publics neuchâtelois (TransN) | 64, Transports urbains delémontains (TUD) | 65, Transports Vallée de Joux, Yverdon-les-Bains, Saint croix (Travys) | 66, Trasporti Pubblici Luganesi (TPL) | 67, Verkehrbetriebe Zürich (VBZ) | 68, Verkehrsbetriebe Biel (TpB) | 69, Verkehrsbetriebe Davos (VBD) | 70, Verkehrsbetriebe Glattal AG (vbg) | 71, Verkehrsbetriebe Luzern (vbl) | 72, Verkehrsbetriebe Schaffhausen (vbsh) | 73, Verkehrsbetriebe St. Gallen (VBSG) | 74, Verkehrsbetriebe Biel (TpB) Gallen (VBSG) | 74, Verkehrsbetriebe Zürichsee un Oberland (VZO) | 75, Vevey-Montreux-Chillon-Villeneuve (VMCV) | 76, WilMobil AG (Wilmob) | 77, Zugerland Verkehrsbetriebe AG (zvb) | 78, Regionalverkehr Bern-Solothurn (RBS) | 79, Other

1.1 If other *(specify)*

………………………..

2. When did you start working there? *Date:*

☐☐.☐☐☐☐

Month .Year

3. Do you work or have you worked there full time (100%)?

☐ Yes ☐ No

3.1 If not, at what rate do you or did you work there?

☐☐ %

4. How many bus models have you driven for this company?

☐☐ model(s)

Number from 1 to 10

According to the number indicated, display the following text.

If 1 new model: Can you tell us more about this bus?

If >1 new models: Can you tell us more about these buses, starting with the latest?

Displaying block questions

5. Using the search tool below, please select the bus model (one answer only).

Filtrer :

Entreprise

Marque

Modèle

Boîte de vitesse

Gabarit

Motorisation

Show 10 **entries** **Search:**

Entreprise	Marque	Modèle	Année de mise en service (Suisse)	Année de mise en service (Entreprise)	Boîte de vitesse	Gabarit	Motorisation
Transports publics fribourgeois (TPF)	Berliert	EH 100 S	1962	1963	Continue/sans (avant/arrière)	Standard & midi	Trolley
Stadtbus Winterthur (SW)	Berna	4 GTP	1965	1965	Continue/sans (avant/arrière)	Articulé	Trolley
Verkehrsbetriebe Schaffhausen (vbsh)	Berna	4 GTP	1965	1966	Continue/sans (avant/arrière)	Articulé	Trolley

☐ I can't find the bus model ☐ I don't know

6.1. If "I can't find the bus model". What is the model of the bus you currently drive or have driven most often in this company?

List currently being updated (given as an example)

1, Alexander Denis | 2, Berliert | 3, Berna | 4, Beulas| 5, BOVA/ VDL | 6, BredaMenarinibus | 7, Büssing | 8, FBW | 9, FHS | 10, FIAT | 11, Ford | 12, Hess | 13, Heuliez | 14, Irisbus-Iveco | 15, Irizar | 16, K-Bus / Kutsenits | 17, Magirus-Deutz | 18, MAN | 19, Merceds-Benz | 20, NAW | 21, Neoplan | 22, Opel | 23, Renault | 24, Saurer | 25, Scania | 26, Setra | 27, Solaris | 28, Temsa | 29, Van Hool | 30, Vetra | 31, Volvo | 32, Other

6.2. What is the vehicle size?

☐ Mini ☐ Midi and standard (8-15 m) ☐ Articulated ☐ Bi-articulate ☐ Imperial (two-stage)

6.3. What type of engine does the vehicle have?

☐ Combustion engine (diesel, gasoline, gas) ☐ Hybrid ☐ Electric ☐ Trolley

6.4. What type of gearbox does the vehicle have?

☐ Manual ☐ Semi-automatic ☐ automatic ☐ Robotic ☐ Without gearbox or Continuous (forward/reverse)

7. How often do you or did you drive this vehicle? Please indicate the number of half-days per week (to determine driving rate).

☐ 1 ☐2 ☐ 3 ☐4 ☐ 5 ☐6 ☐7 ☐8 ☐9 ☐10 ☐ 11 ☐12 ☐ 13 ☐14

☐ Less than half a day

8. When did you start driving this bus?

☐☐ . ☐☐☐☐

Month Year

9. When did you stop driving it?

☐☐ . ☐☐☐☐

Month Year

10. What type of service do you perform or have you performed with this bus?

☐ Urban ☐ Regional ☐ Mixed

10.1 If mixed, what percentage do you estimate drives or has driven this vehicle on urban routes? (Indicate percentage rate)

☐☐ %

Repeat Vehicle block

Questionnaire on driver station ergonomics and comfort

We're now going to ask you a few questions about the ergonomics and comfort of the driver's cab.
Please think about the bus model you have driven most often in the last 12 months.

Which bus model have you driven most often in the last 12 months?

Using the search tool below, please select the bus model.

Filtrer :

Entreprise

Marque

Modèle

Boîte de vitesse

Gabarit

Motorisation

Show 10 **entries** **Search:**

Entreprise	Marque	Modèle	Année de mise en service (Suisse)	Année de mise en service (Entreprise)	Boîte de vitesse	Gabarit	Motorisation
Transports publics fribourgeois (TPF)	Berliert	EH 100 S	1962	1963	Continue/sans (avant/arrière)	Standard & midi	Trolley
Stadtbus Winterthur (SW)	Berna	4 GTP	1965	1965	Continue/sans (avant/arrière)	Articulé	Trolley
Verkehrsbetriebe Schaffhausen (vbsh)	Berna	4 GTP	1965	1966	Continue/sans (avant/arrière)	Articulé	Trolley

1. Seat comfort

1. On a scale of 1 to 10, how would you rate the overall comfort of the seat? (1 = very uncomfortable / 10 = perfect)

☐ 1 ☐ 2 ☐ 3 ☐ 4 ☐ 5 ☐ 6 ☐ 7 ☐ 8 ☐ 9 ☐ 10

2. Is the seat adjustable to your body type?

☐ Yes, perfectly ☐ Yes, but with limited adjustment ☐ No, it's difficult to adjust

☐ No, it's not adjustable

3. What settings are available to adapt your seat to your needs?

- Height adjustment : ☐ Yes☐ No

- Depth adjustment : ☐ Yes☐ No
- Lumbar adjustment :☐ Yes☐ No
- Back adjustment : ☐ Yes☐ No
- Head adjustment : ☐ Yes☐ No

4. Is the seatbelt comfortable and properly positioned?

☐ Yes, all the time ☐ Yes, sometimes ☐ No, it's uncomfortable

5. Does the seat cushioning system work properly?

☐ Yes, always ☐ No, never ☐ Don't know

6. Do you notice any wear on the seat?

☐ Yes, significantly ☐ Yes, slightly ☐ No, it's in good condition

7. On a scale from 1 to 10, how much does seat wear affect your comfort? (1 = not at all / 10 = very much)

☐ 1 ☐ 2 ☐ 3 ☐ 4 ☐ 5 ☐ 6 ☐ 7 ☐ 8 ☐ 9 ☐ 10

2. Temperature and ventilation

1. Does the seat have an integrated ventilation system?

☐ Yes, heating only ☐ Yes, heating and cooling ☐ No ☐ Don't know

2. Are your feet cold or too hot in winter?

☐ Cold ☐ Too hot ☐ Correct temperature

3. Do you feel draughts in the cabin?

☐ Yes, often ☐ Yes, sometimes ☐ No, never

4. Is the ventilation easy to adjust?

☐ Yes, often ☐ Yes, sometimes ☐ No, never

5. Regarding air conditioning, would you like :

☐ More efficient air conditioning ☐ More air conditioning ☐ Less air conditioning ☐ Better individual control

3. Driving position and physical pain

1. Is the steering wheel position adapted to your morphology?

☐ Yes, perfectly ☐ Yes, but with some difficulty ☐ No, it's uncomfortable

2. On a scale of 1 to 10, how would you rate the physical effort required to manoeuvre the steering wheel on a daily basis? (1 = very easy / 10 = very tiring)

☐ 1 ☐ 2 ☐ 3 ☐ 4 ☐ 5 ☐ 6 ☐ 7 ☐ 8 ☐ 9 ☐ 10

3. Do you feel any pain while driving? (multiple choices possible)

☐ Arm pain ☐ Shoulder pain ☐ Back pain ☐ Leg pain

☐ Neck pain ☐ No pain

4. Do you think these pains are related to :

☐ The hardness of the steering wheel ☐ The number of bends or turns ☐ Prolonged posture ☐ Other : ____________________________________

4. Visibility and sound environment

1. On a scale of 1 to 10, how would you rate visibility from your driving position? (1 = very poor / 10 = perfect)

☐ 1 ☐ 2 ☐ 3 ☐ 4 ☐ 5 ☐ 6 ☐ 7 ☐ 8 ☐ 9 ☐ 10

2. Do any accessories interfere with your visibility (screen, tablet, stand, etc.)?

☐ Yes, often ☐ Yes, sometimes ☐ No, never

3. If yes, please rate the following on a scale of 1 to 5 (from "Not at all bothersome" to "Very bothersome"), ranking them from most to least bothersome:

☐ Passenger information display ☐ Management tablet ☐ Phone holder

☐ Reflections/excessive brightness ☐ Positioning of accessories (screen, tablet, etc.)

☐ Notifications or alerts displayed on devices

4. Does the noise level in the cabin bother you?

☐ Yes, often ☐ Yes, sometimes ☐ No, never

5. If yes, please rate the following on a scale of 1 to 5 (from "Not at all bothersome" to "Very bothersome"), ranking them from most to least bothersome:

☐ Passengers talking loudly ☐ Passengers using their phones

☐ Mechanical bus noises ☐ Audible safety signals (beeps, alarms, etc.)

5. Customer relations

1. Would you like to be more isolated from customers?

☐ Yes, in most situations☐ Yes, but only in certain situations☐ No, I prefer to stay in contact with customers

2. Does direct contact with customers interfere with your concentration?

☐ Yes, often ☐ Yes, sometimes ☐ No, never

6. Vehicle condition

1. Does the age of your vehicle have an impact on your driving comfort?

☐ Yes, often ☐ Yes, sometimes ☐ No, never

2. Do you experience technical or comfort problems with older vehicles?

☐ Yes, often ☐ Yes, sometimes ☐ No, never

If yes, please specify:

☐ ____

☐ ____

11. Have you worked for another public transport company?

☐ **Yes** ☐ **No**

If yes to Q11: Former company

11.1 In which company did you previously work?

3, Aargau Verkehr (AVA) | 4, Busbetrieb Aarau AG (BBA) | 5, Aare Seeland mobil | 6, Autilinea Mendriense (AMSA) | 7, Autolinee Bleniesi SA (ABL) | 8, Auto AG Group (AAGR) | 9, Auto AG Schwyz | 10, Auto Ag Uri | 11, Autobetrieb Sernftal | 12, Autobetrieb Weesen-Amden (AWA) | 13, Autobus AG Liestal (AAGL) | 14, Autokurse Oberthurgau (AOT) | 15, Autolinee Regionali Luganesi (arl) | 16, Automobilverkehr Frutigen-Adelboden (AFA) | 17, Baselland Transport (BLT) | 18, Basler Verkehrsbetriebe (BVB) | 19, Bernmobil | 20, Busbetrieb Seetal Freiamt (BSF) | 21, BUS Ostschweiz AG (BO) | 22, Bus sédunois | 23, Busbetrieb Grenchen und Umgebung (BGU) | 24, Bus Sarganserland-Werdenberg (BSW) | 25, Busbetrieb Linchtensteig-Wattwil-Ebnat-Kappel (blwe) | 26, Busbetrieb Olten Gösgen Gäu (BOGG) | 27, Busbetrieb Solothurn und Umgebung (BSU) | 28, Busland AG Langnau Burgdorf (BLS) | 29, Chemin de fer du jura | 30, Compagnie de Chemin de fer et d'autobus Sierre-Montana-Crans SA (SMC) | 31, Engadin Bus | 32, Ferrovie Autolinee Regionali Ticinesi (FART) Locarno | 33, GEM'Bus | 34, Genève tour (G-tour) | 35, Globe Limo | 36, Odier Excursion | 37, Pfosi Arosa Bus AG | 38, PostAuto Schweiz AG (Carpostal) | 39, Ratpdev | 40, RDTA | 41, Regiobus AG Gossau (Regio) | 42, Regionalbus Lenzburg(RBL) | 43, Regionale Verkehrsbetriebe Baden-Wettingen (RVBW) | 44, Regionale Verkehrsbetriebe Schaffhausen RVSH | 45, Rottal Auto AG | 46, SBB-Bus Zofingen/Reiden | 47, Schneider Busbetriebe | 48, Seebus | 49, Sihltal Zürich Uetliberg Bahn AG (SZU) | 50, Stadtbus Chur | 51, Stadtbus Frauenfeld | 52, Stadtbus Kreuzlingen | 53, Stadtbus Winterthur (SW) | 54, STI Bus AG | 55, Tellbus Altdorf-Luzern | 56, Transports de la région Morges-Bière-Cossonay (MBC) | 57, Transports Martigny Région (TMR) | 58, Transports publics de la région lausannoise (tl) | 59, Transports publics de la région nyonnaise (tpn) | 60, Transports publics du Chablais (TPC) | 61, Transports publics fribourgeois (TPF) | 62, Transports publics genevois (TPG) | 63, Transports publics neuchâtelois (TransN) | 64, Transports urbains delémontains (TUD) | 65, Transports Vallée de Joux, Yverdon-les-Bains, Saint croix (Travys) | 66, Trasporti Pubblici Luganesi (TPL) | 67, Verkehrbetriebe Zürich (VBZ) | 68, Verkehrsbetriebe Biel (TpB) | 69, Verkehrsbetriebe Davos (VBD) | 70, Verkehrsbetriebe Glattal AG (vbg) | 71, Verkehrsbetriebe Luzern (vbl) | 72, Verkehrsbetriebe Schaffhausen (vbsh) | 73, Verkehrsbetriebe St. Gallen (VBSG) | 74, Verkehrsbetriebe Biel (TpB) Gallen (VBSG) | 74, Verkehrsbetriebe Zürichsee un Oberland (VZO) | 75, Vevey-Montreux-Chillon-Villeneuve (VMCV) | 76, WilMobil AG (Wilmob) | 77, Zugerland Verkehrsbetriebe AG (zvb) | 78, Regionalverkehr Bern-Solothurn (RBS) | 79, Other

11.2 When did you start working for this company?

☐☐.☐☐☐☐

Month . Year

11.3 When did you stop working for this company?

☐☐.☐☐☐☐

Month . Year

11.4. Did you work full-time (100%)?

☐ Yes ☐ No

11.5 If not, at what rate did you work?

☐☐ %

Repeat Vehicle block

Repeat block Old company
